# Supplementary material for: Identification and Characterization of Small RNA Markers of Age in the Blow Fly Cochliomyia macellaria (Fabricius) (Diptera: Calliphoridae)
Source: Insects. 2022 Oct 18;13(10):948. doi: 10.3390/insects13100948 (PMC9603907; doi:10.3390/insects13100948)
Supplement: Supplementary file 1 [file insects-13-00948-s001.zip › Supplemental Data/Hjelmen_et_al_Supplementary_Cmac_miRNA.pdf]

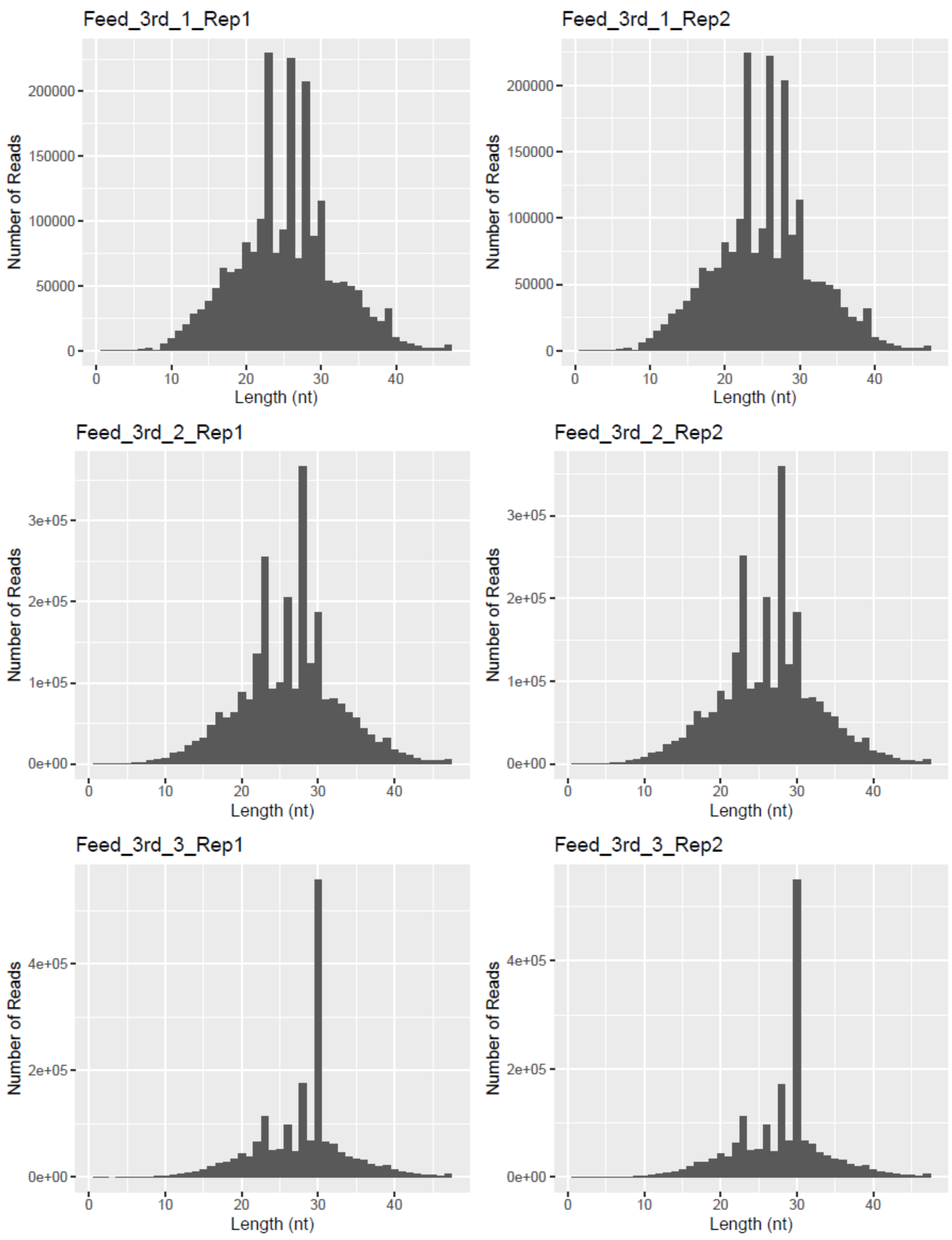

Figure S1: Distribution of number of reads by read length for Feeding 3rd instar samples

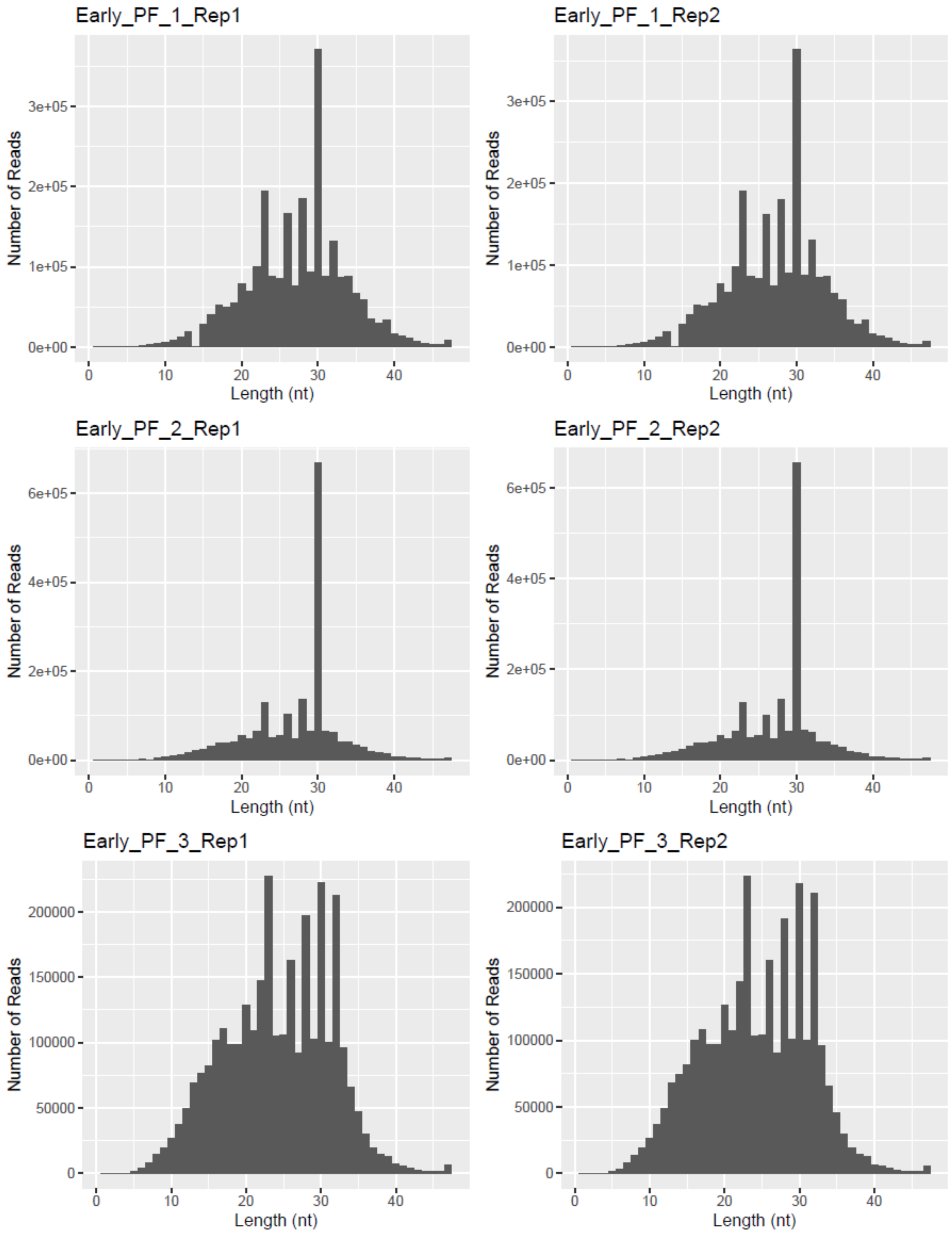

Figure S2: Distribution of number of reads by read length for Early postfeeding samples

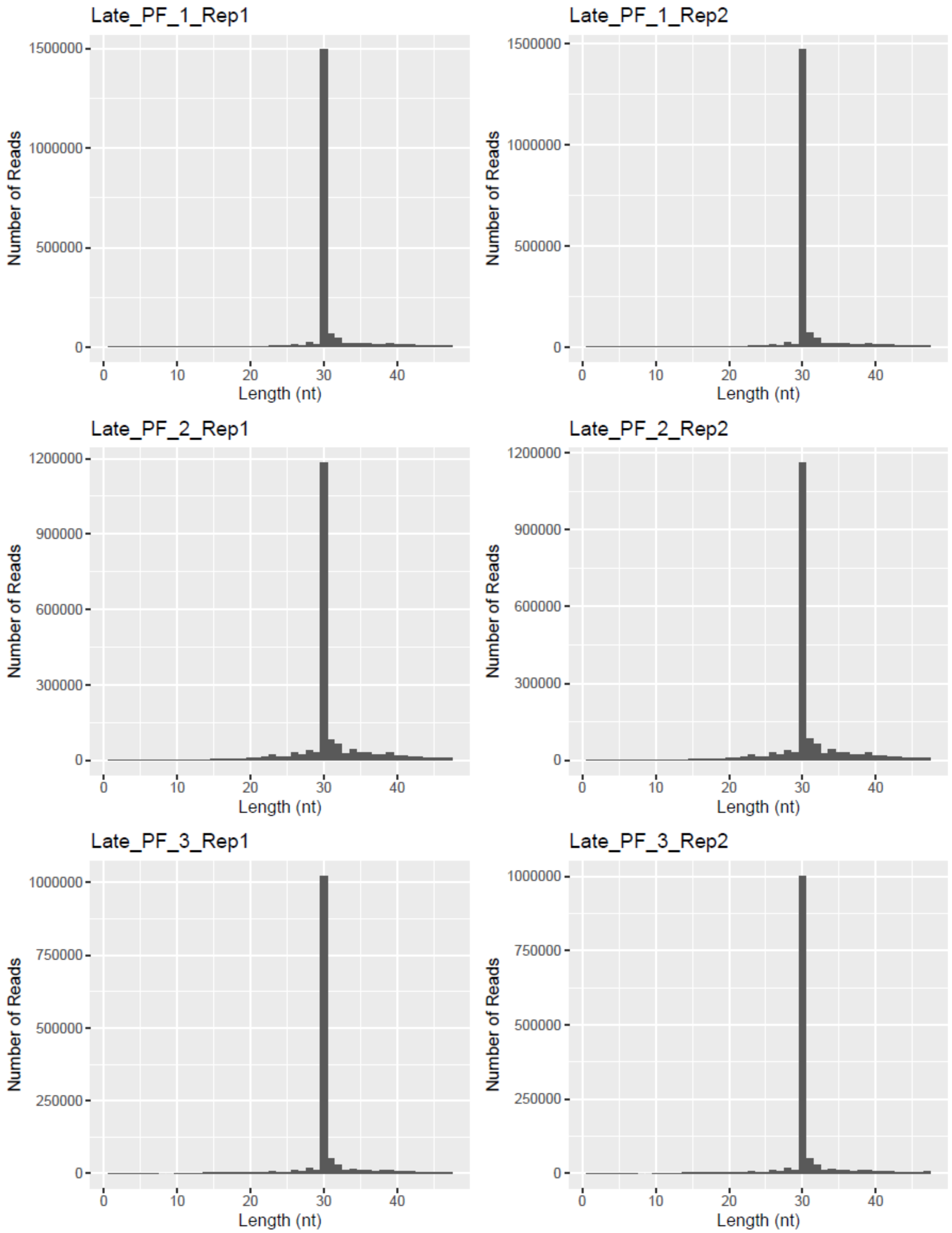

Figure S3: Distribution of number of reads by read length for Late postfeeding samples

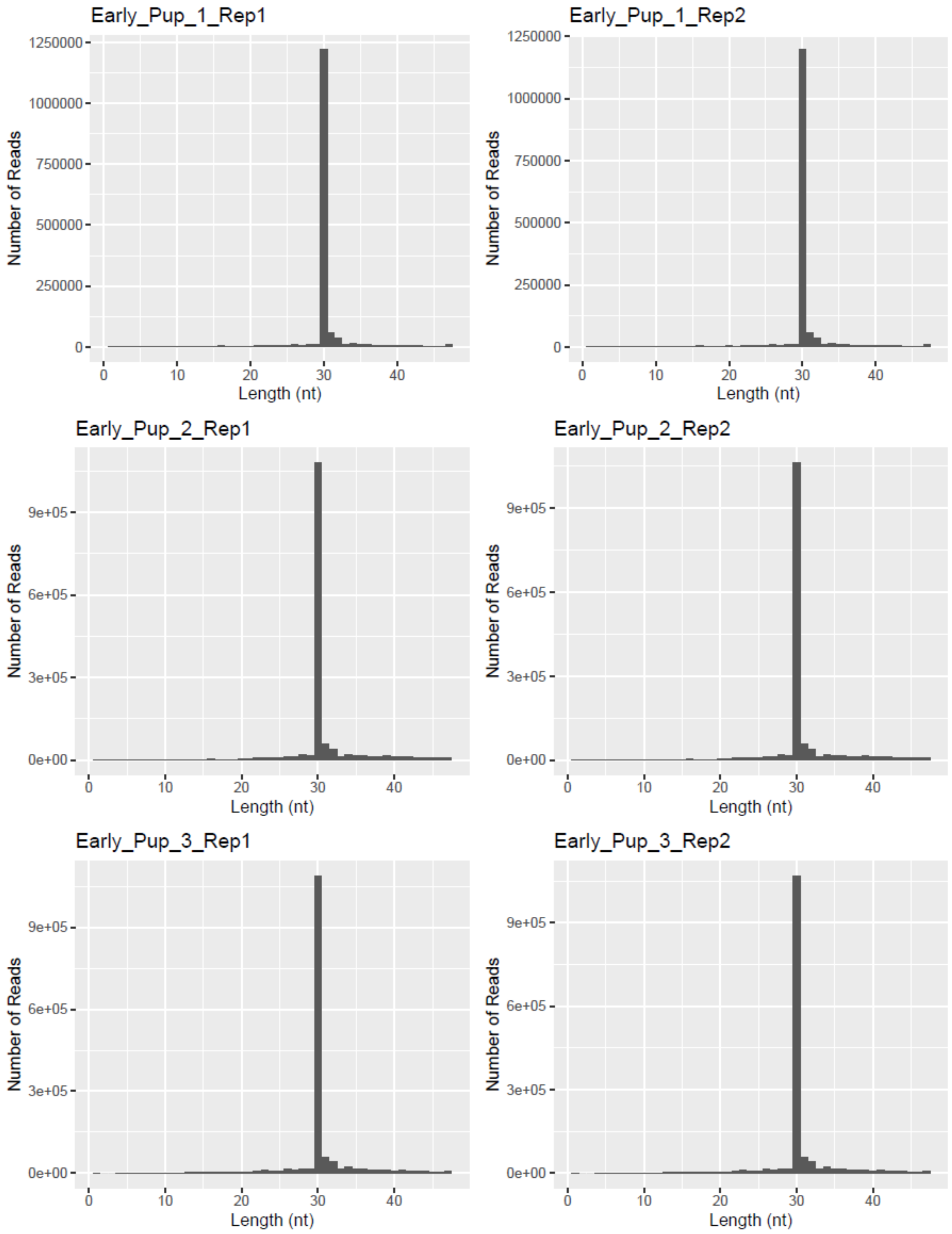

Figure S4: Distribution of number of reads by read length for Early intrapuparial samples

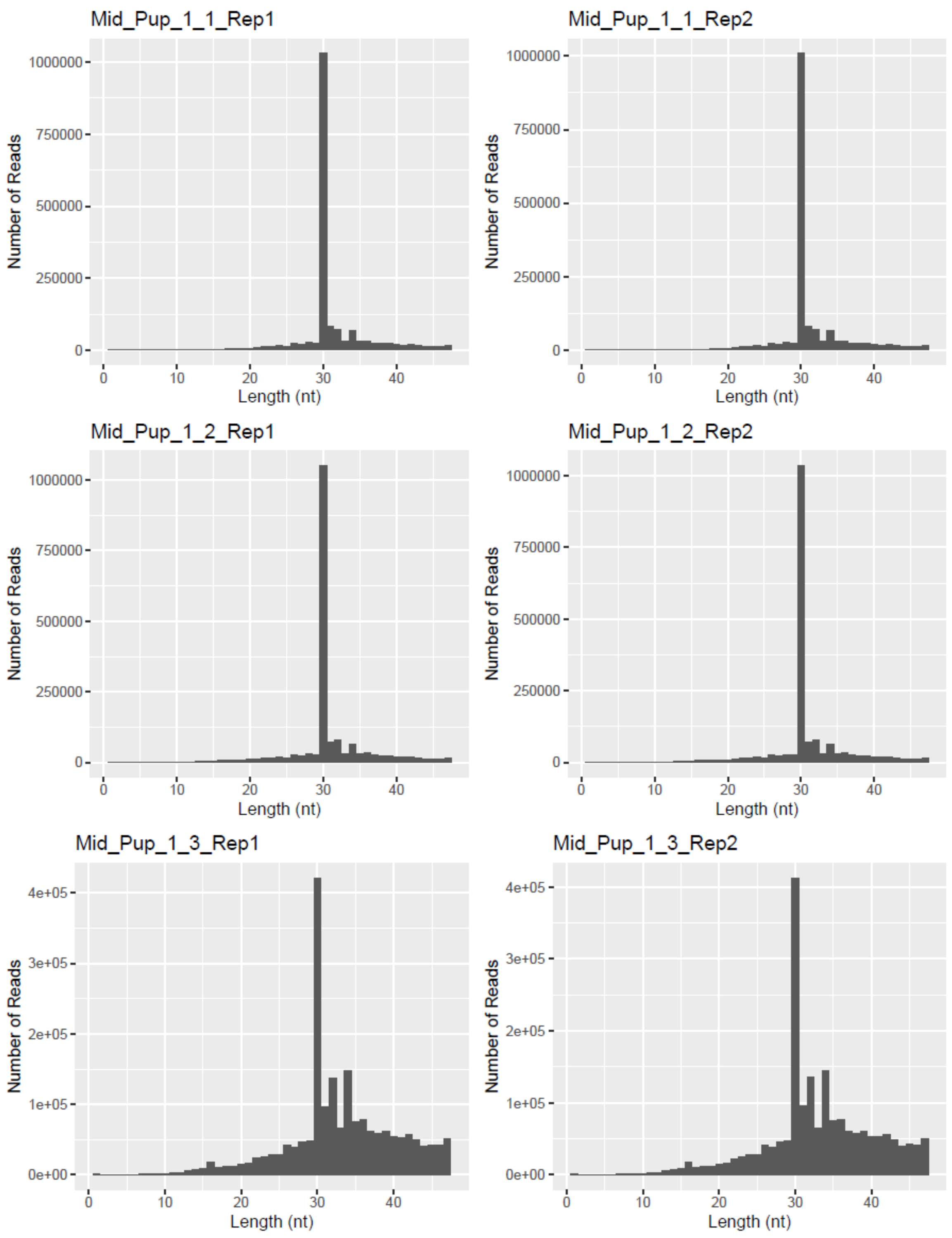

Figure S5: Distribution of number of reads by read length for Mid intrapuparial 1 samples

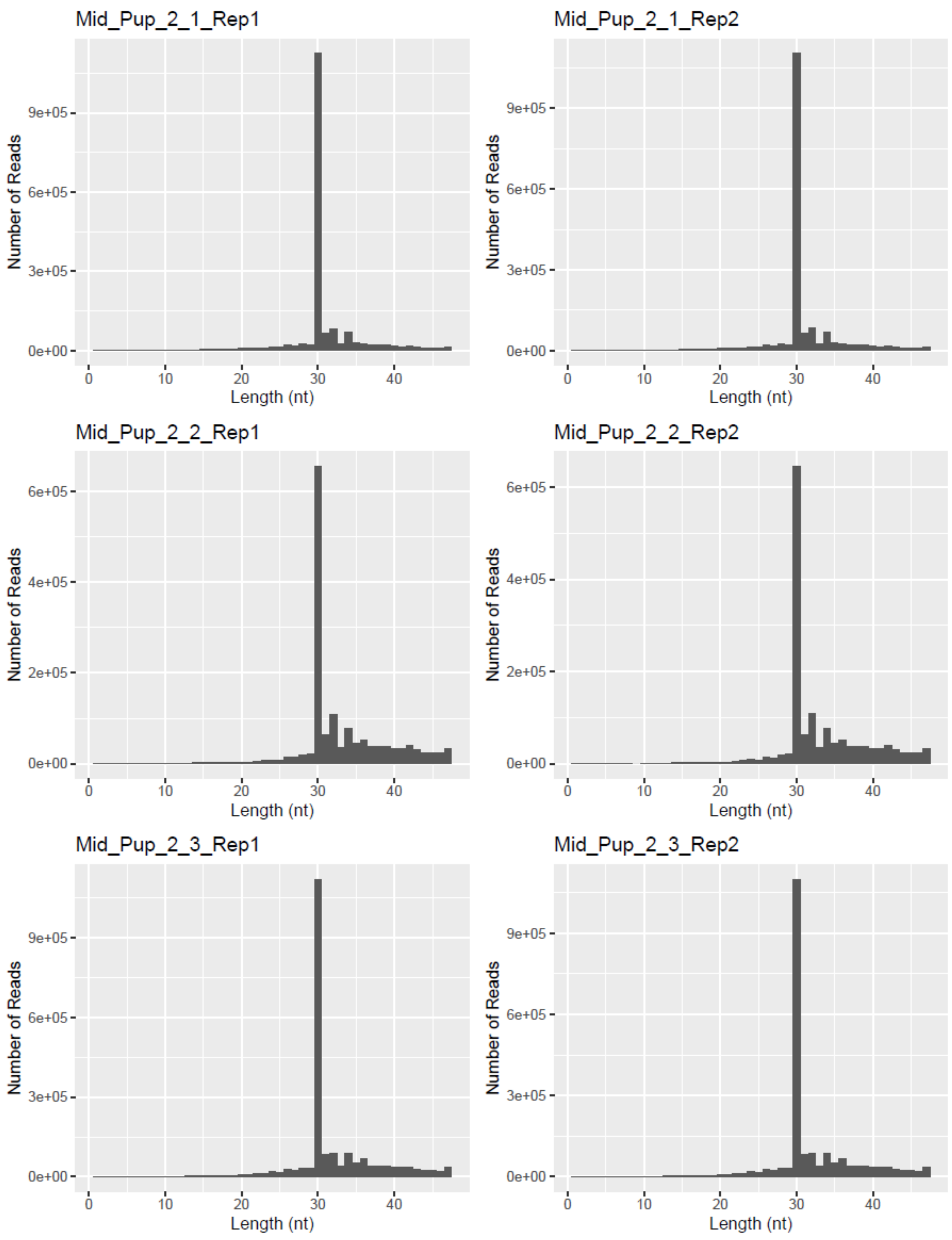

Figure S6: Distribution of number of reads by read length for Mid intrapuparial 2 samples

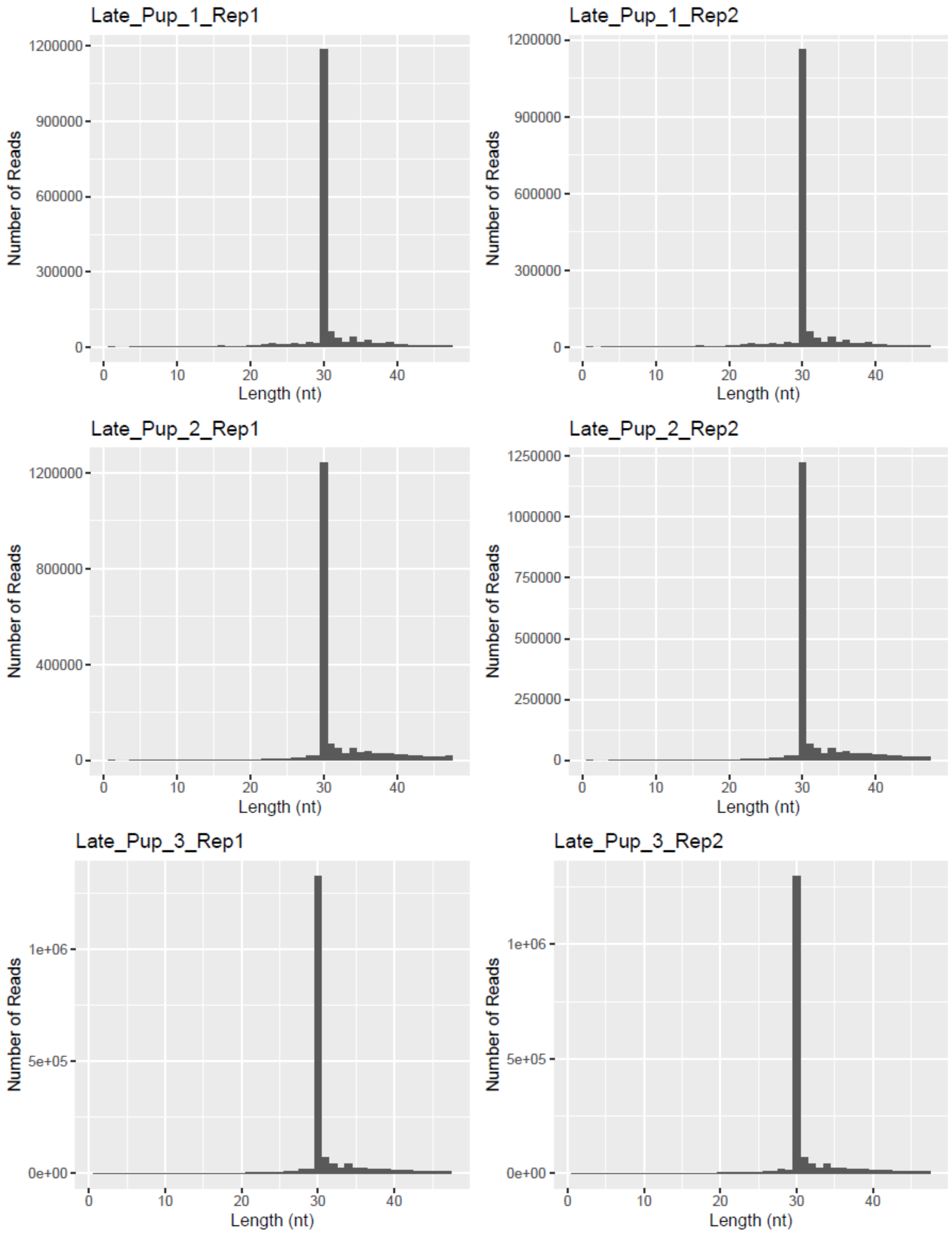

Figure S7: Distribution of number of reads by read length for Late intrapuparial samples

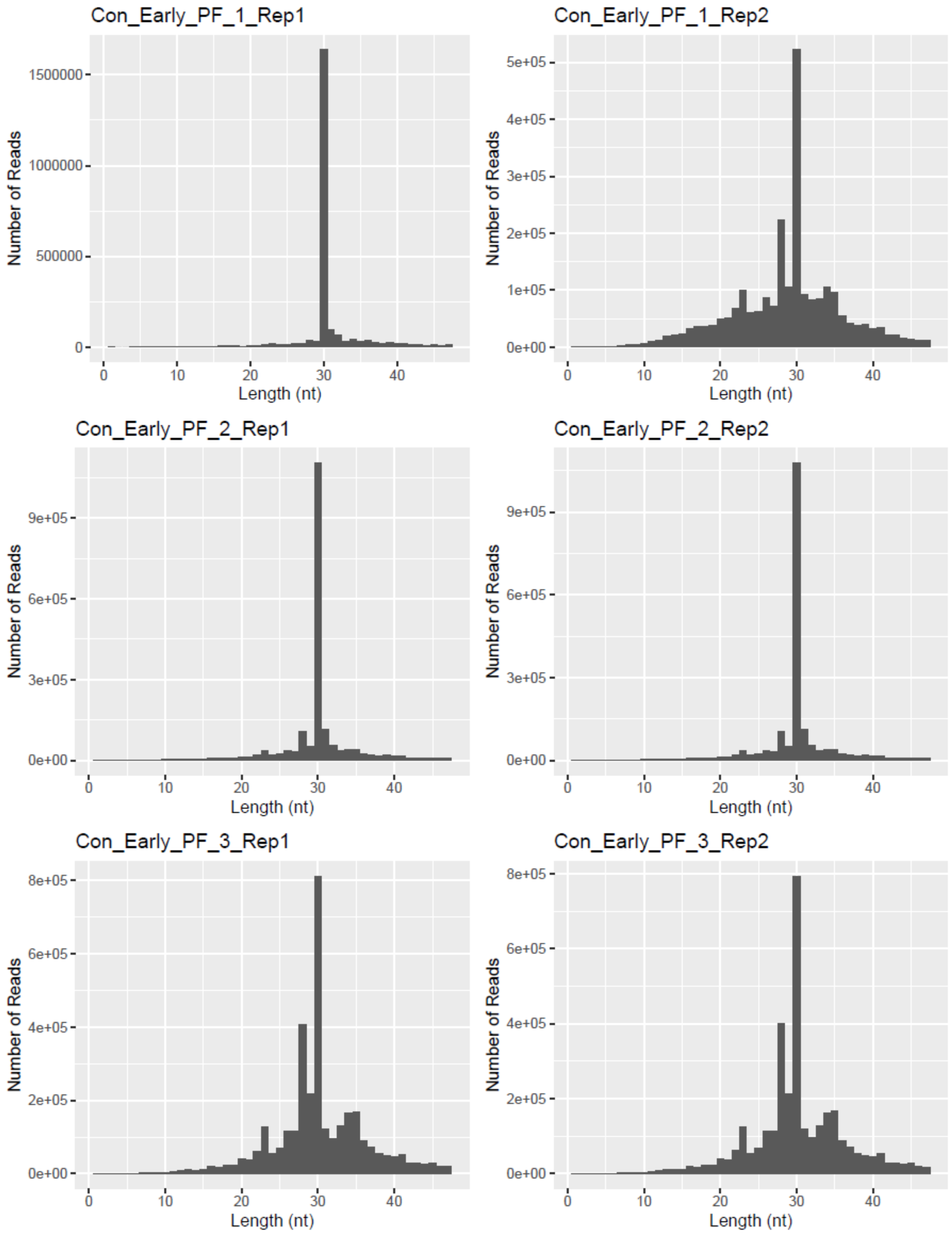

Figure S8: Distribution of number of reads by read length for Control early postfeeding samples

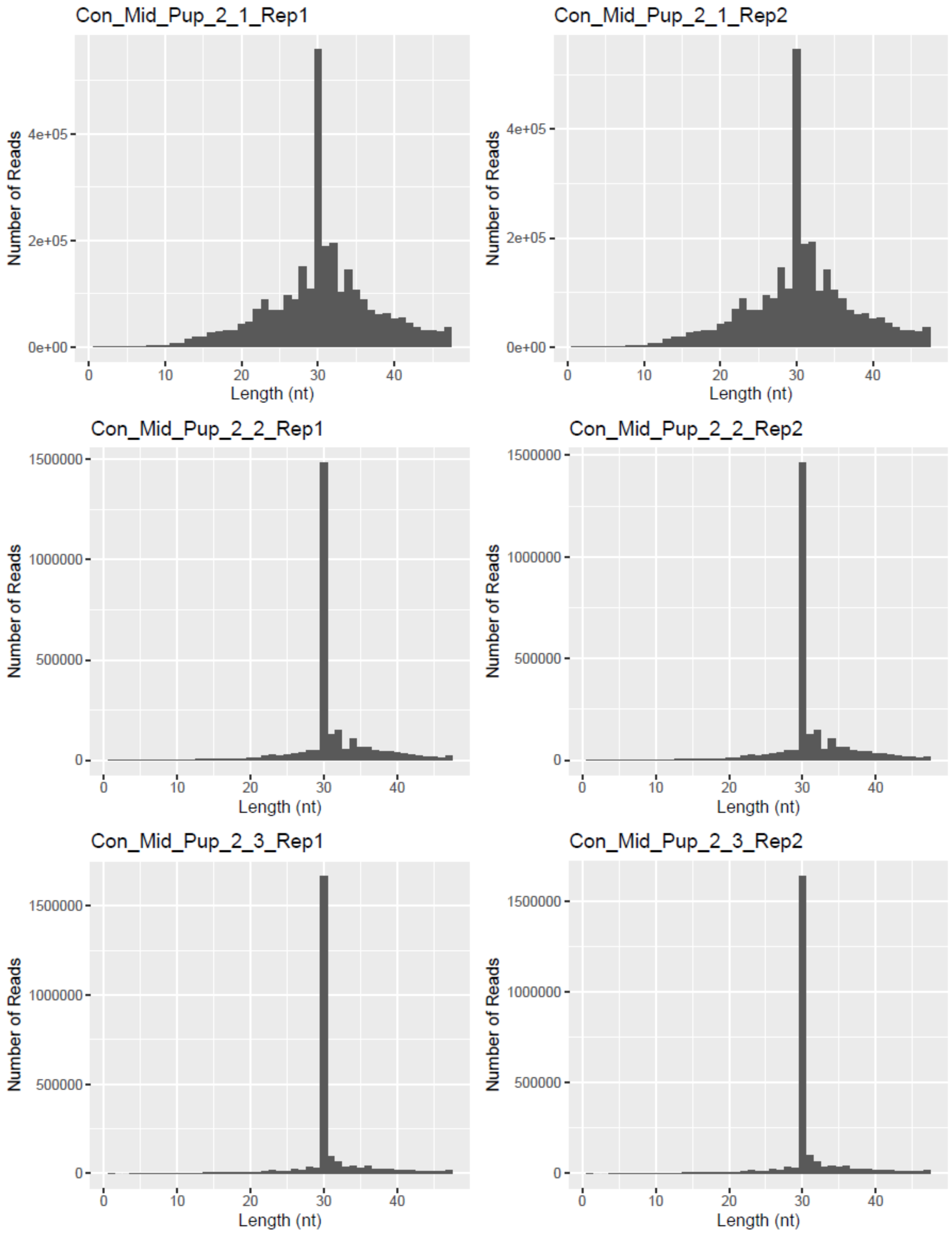

Figure S9: Distribution of number of reads by read length for Control mid-intrapuparial 2 samples

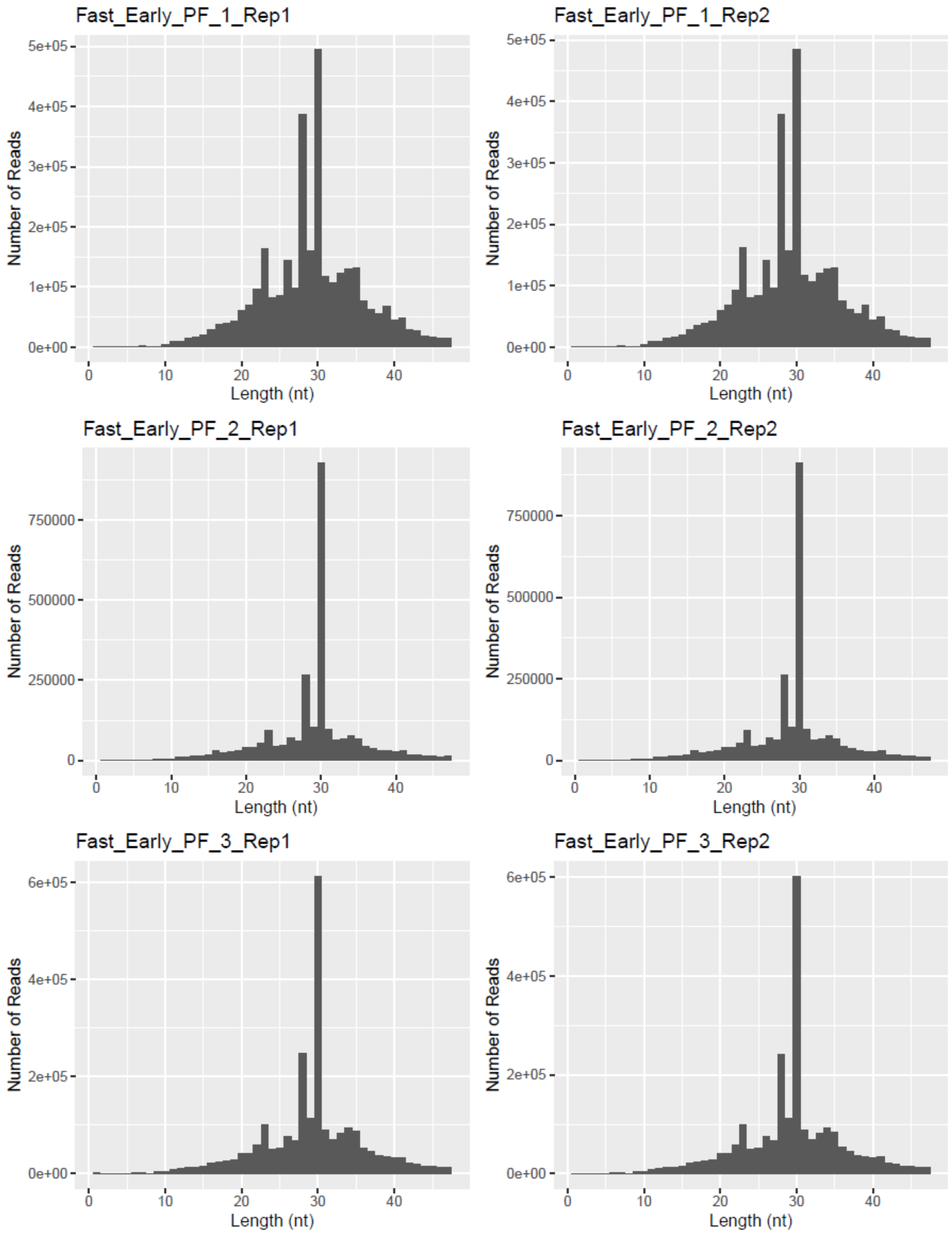

Figure S10: Distribution of number of reads by read length for Fast Early postfeeding samples

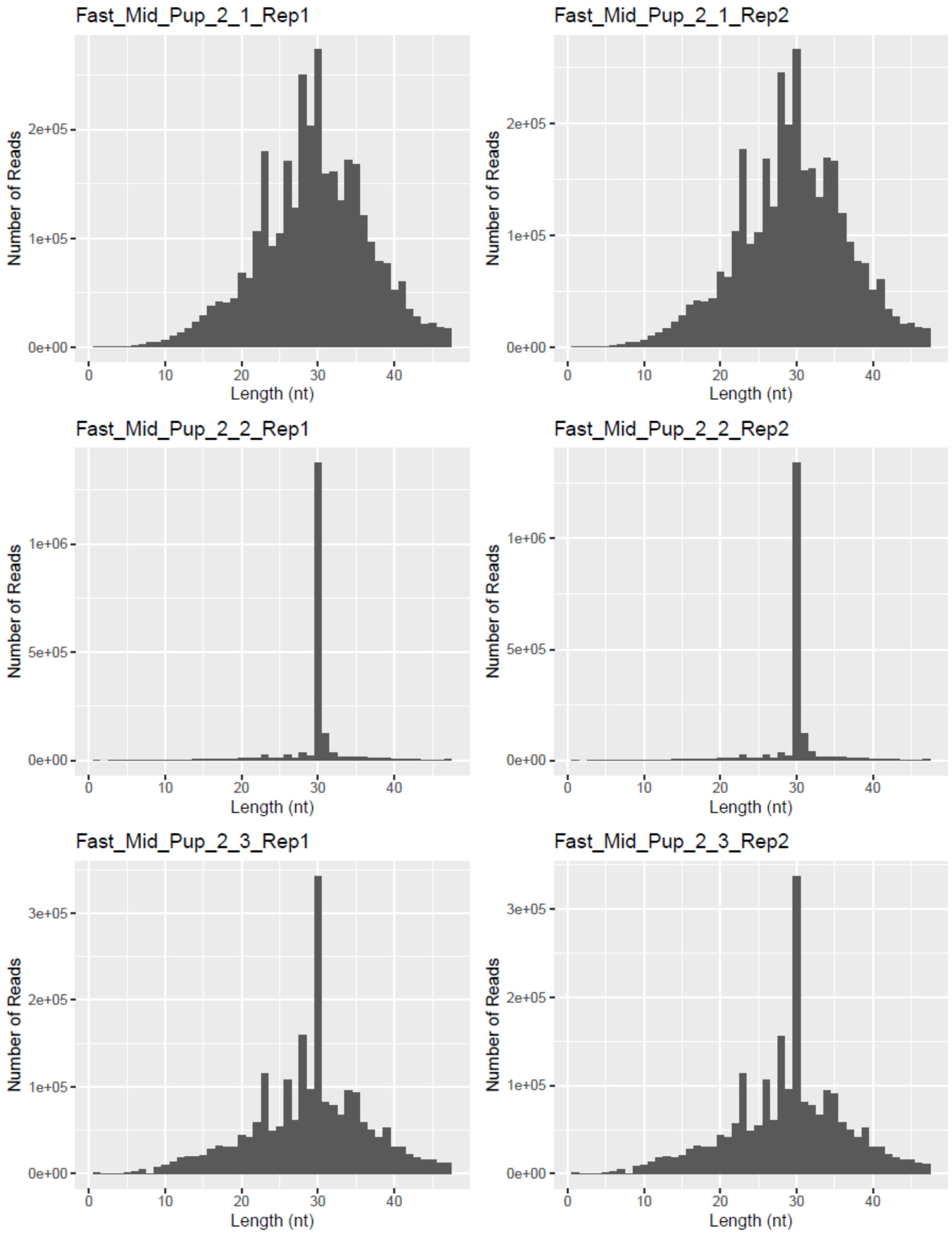

Figure S11: Distribution of number of reads by read length for Fast mid intrapuparial 2 samples

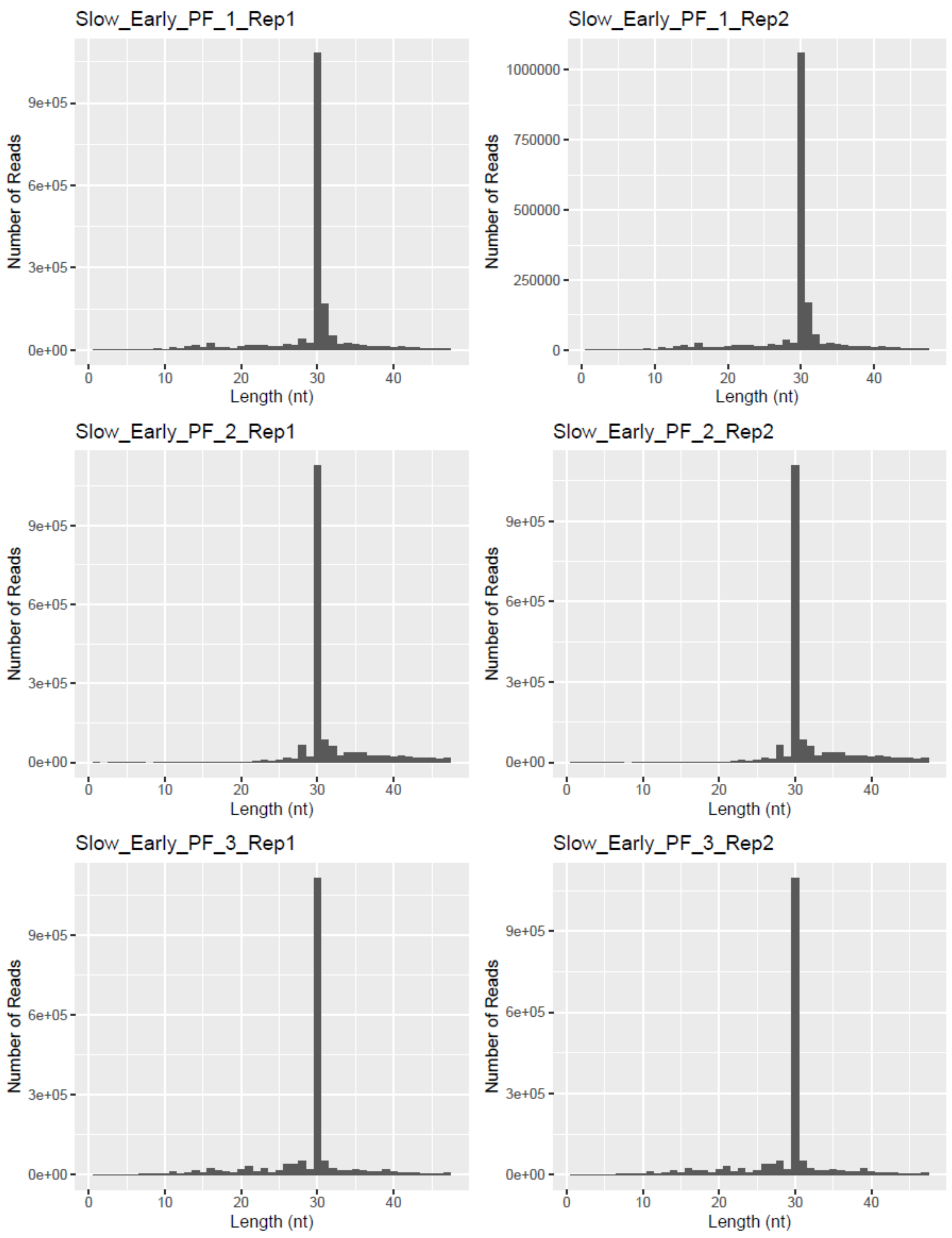

Figure S12: Distribution of number of reads by read length for Slow early postfeeding samples

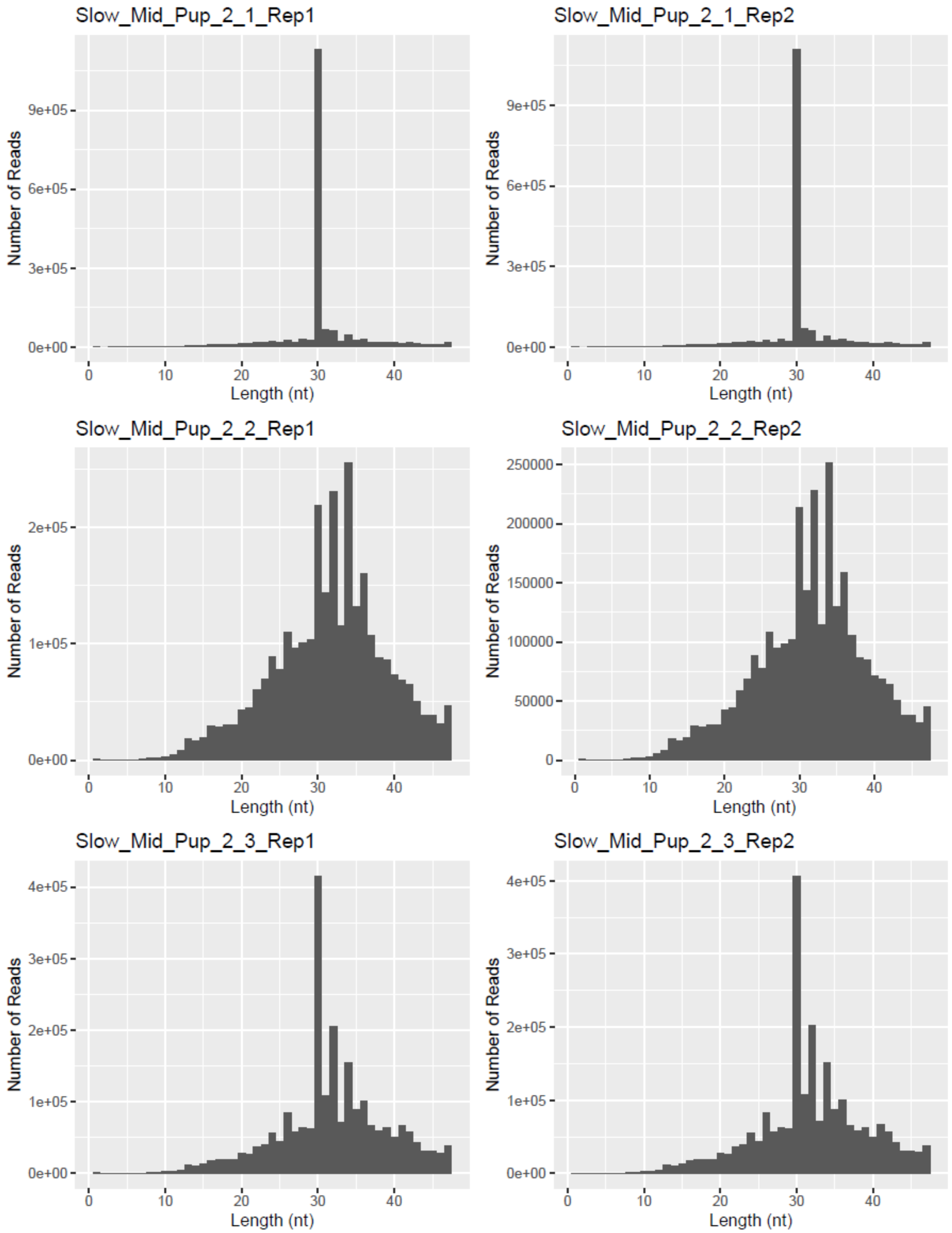

Figure S13: Distribution of number of reads by read length for Slow mid intrapuparial 2 samples

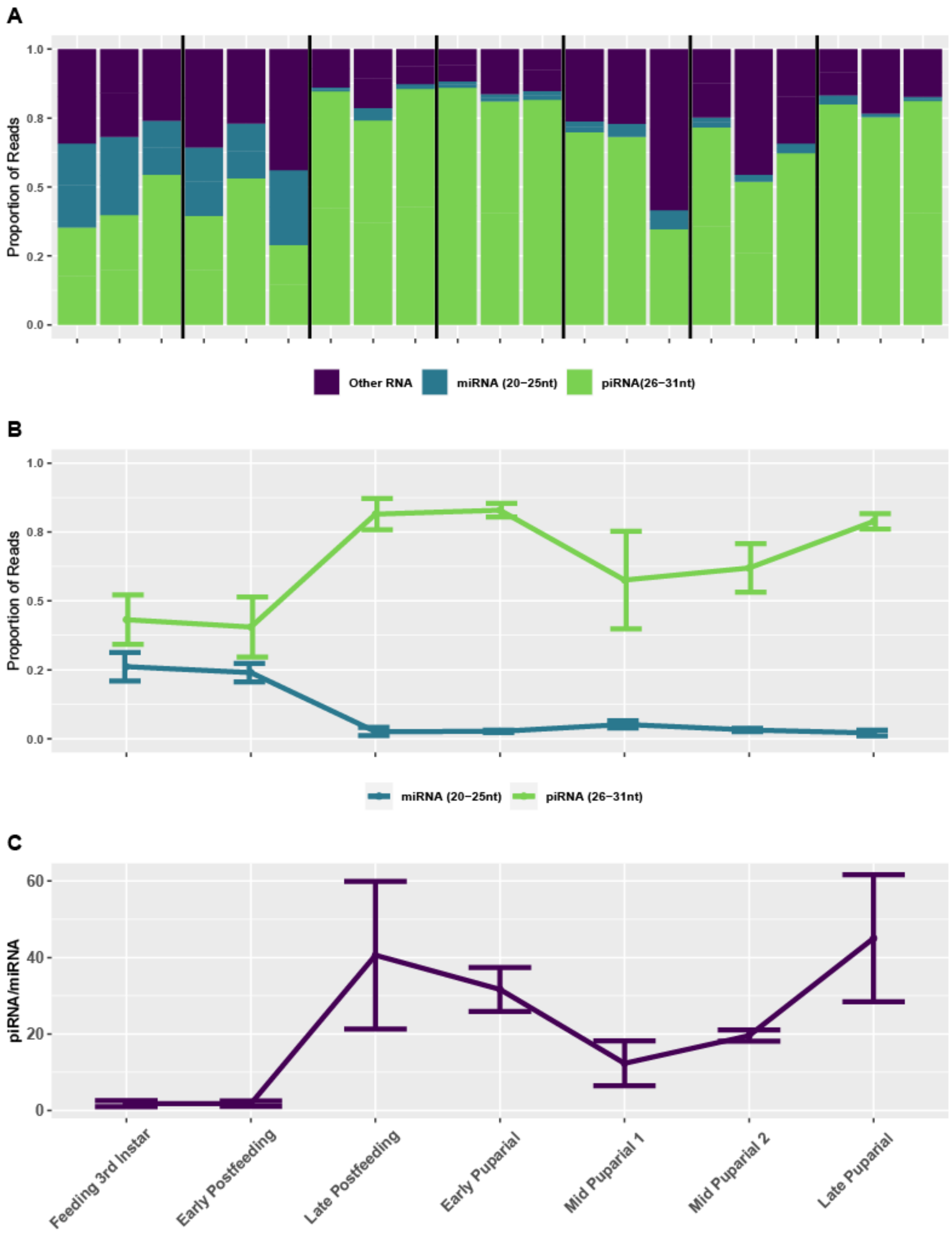

Figure S14: Proportions of RNA which are likely miRNA versus piRNA. Reads likely to be miRNA (20-25nt) and piRNA (26-31nt) were summed and compared to the total number of reads for each sample stage to calculate a proportion of reads. A) Proportion of miRNA (20-25nt), piRNA (26-31nt), and all other RNA from RNA sequences visualized as a stacked bar graph. B). Proportion of miRNA reads (blue) and piRNA reads (green) as they change throughout time. Whiskers represent standard deviation around the mean for each sample.

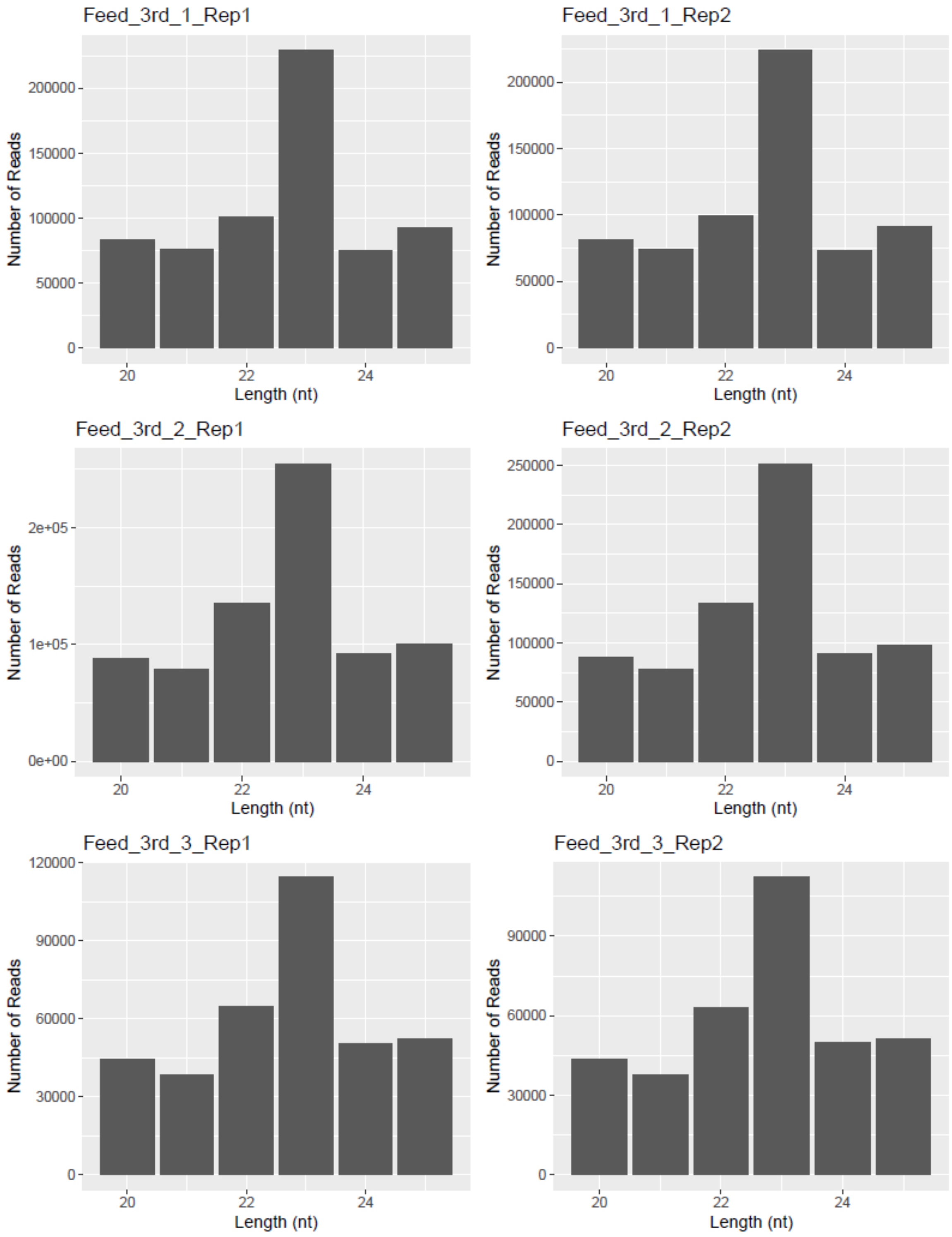

Figure S14: Distribution of number of reads by read length (20-25 nt) for Feeding 3rd instar samples

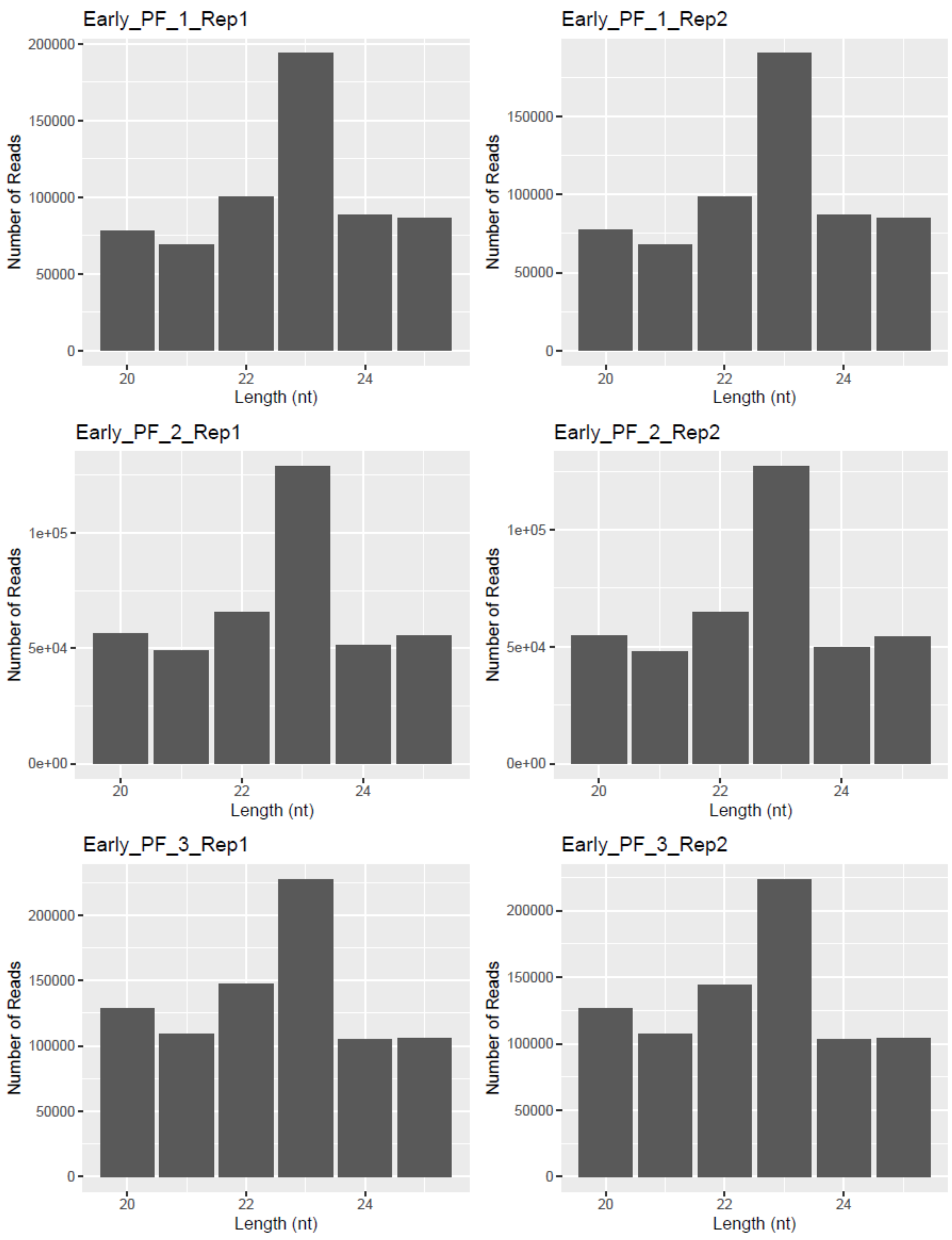

Figure S15: Distribution of number of reads by read length (20-25 nt) for Early postfeeding samples

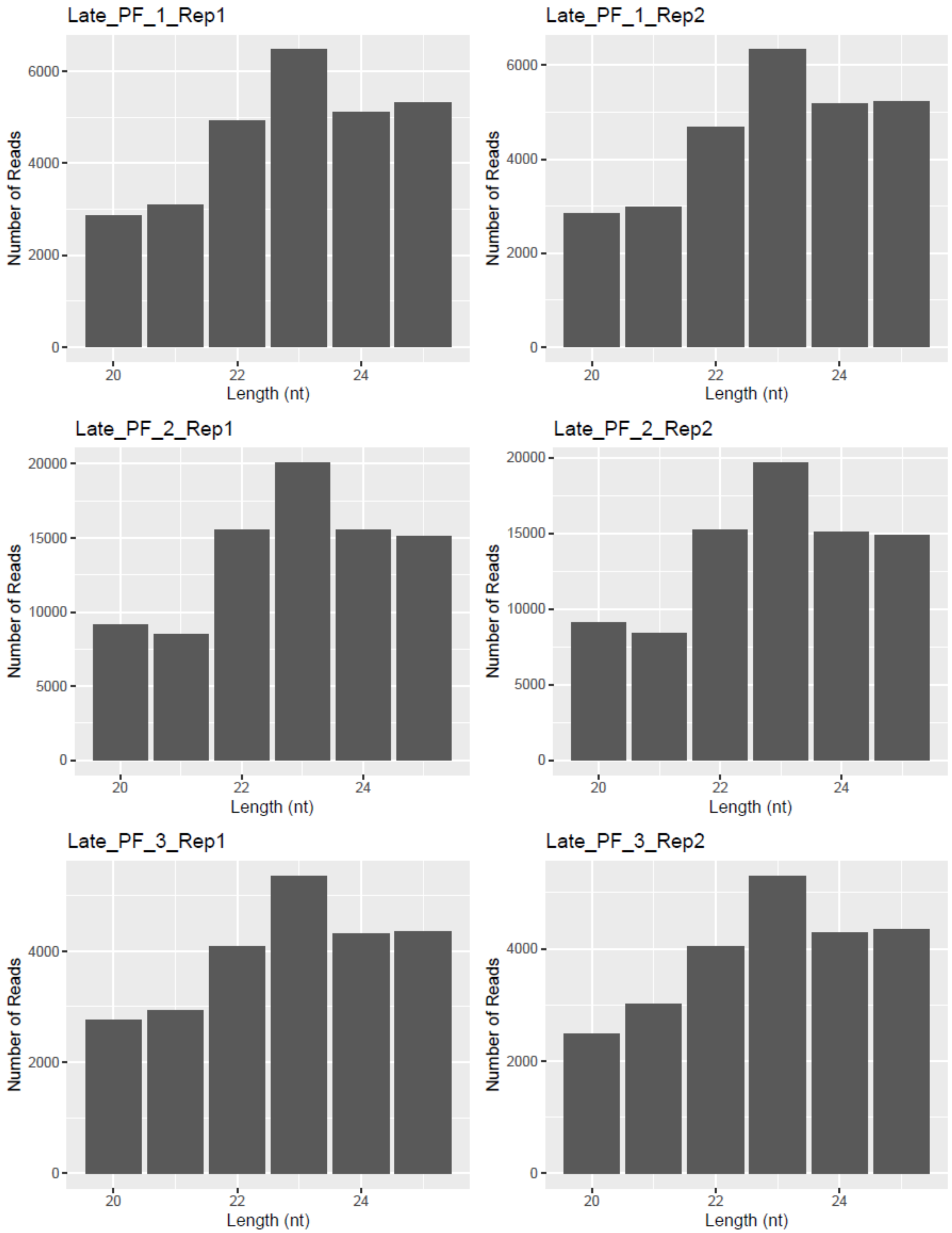

Figure S16: Distribution of number of reads by read length (20-25 nt) for Late postfeeding samples

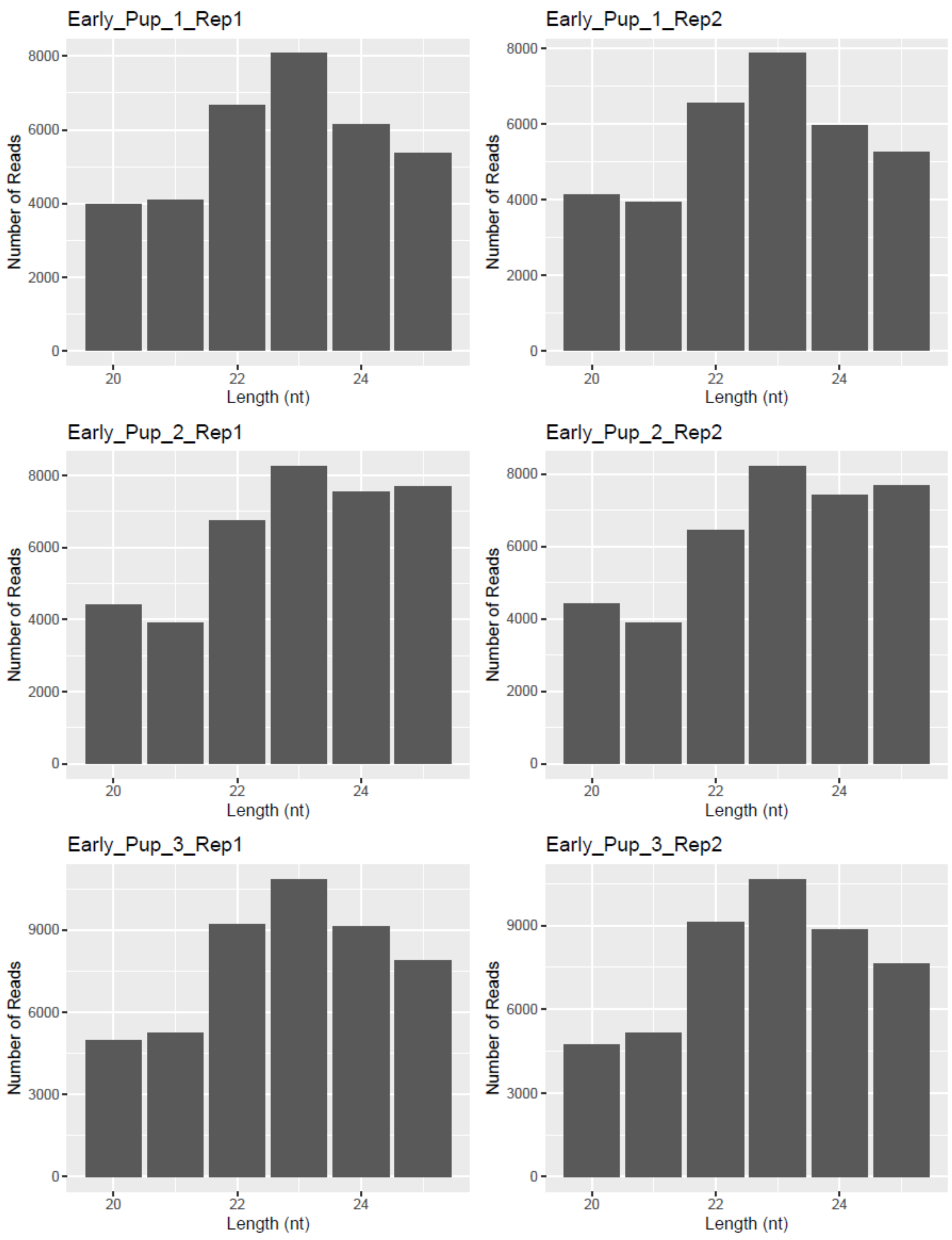

Figure S17: Distribution of number of reads by read length (20-25 nt) for Early intrapuparial samples

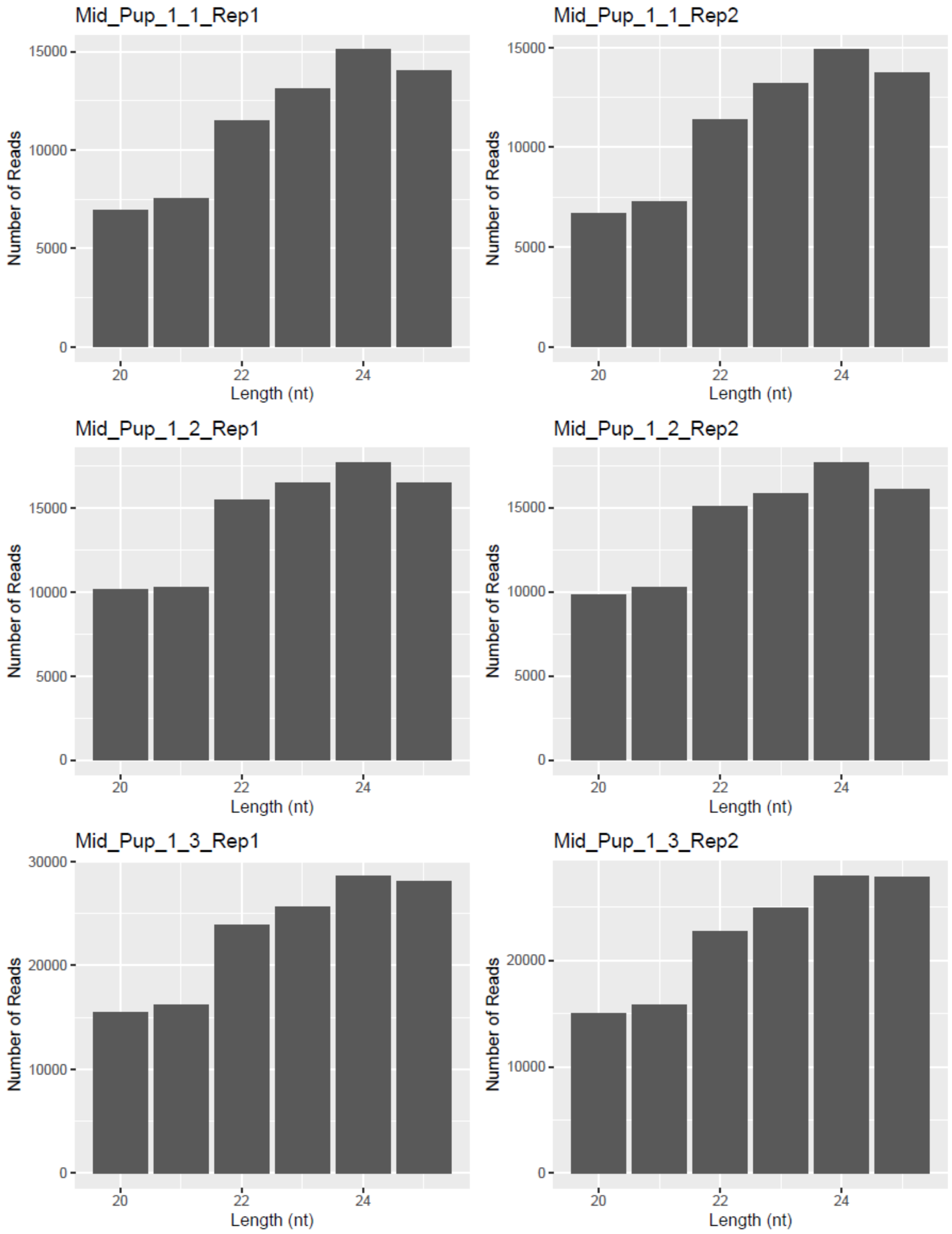

Figure S18: Distribution of number of reads by read length (20-25 nt) for Mid intrapuparial 1 samples

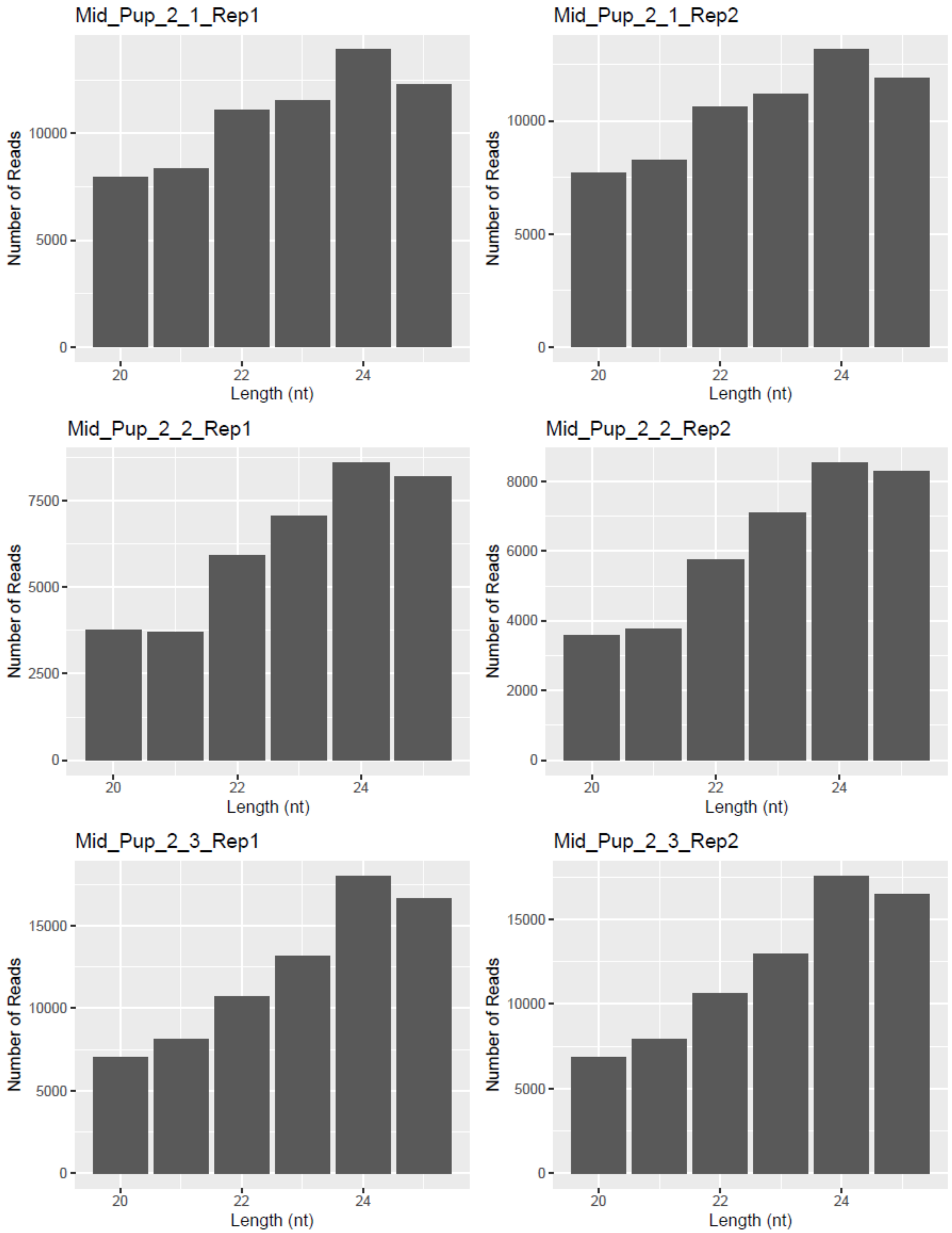

Figure S19: Distribution of number of reads by read length (20-25 nt) for Mid intrapuparial 2 samples

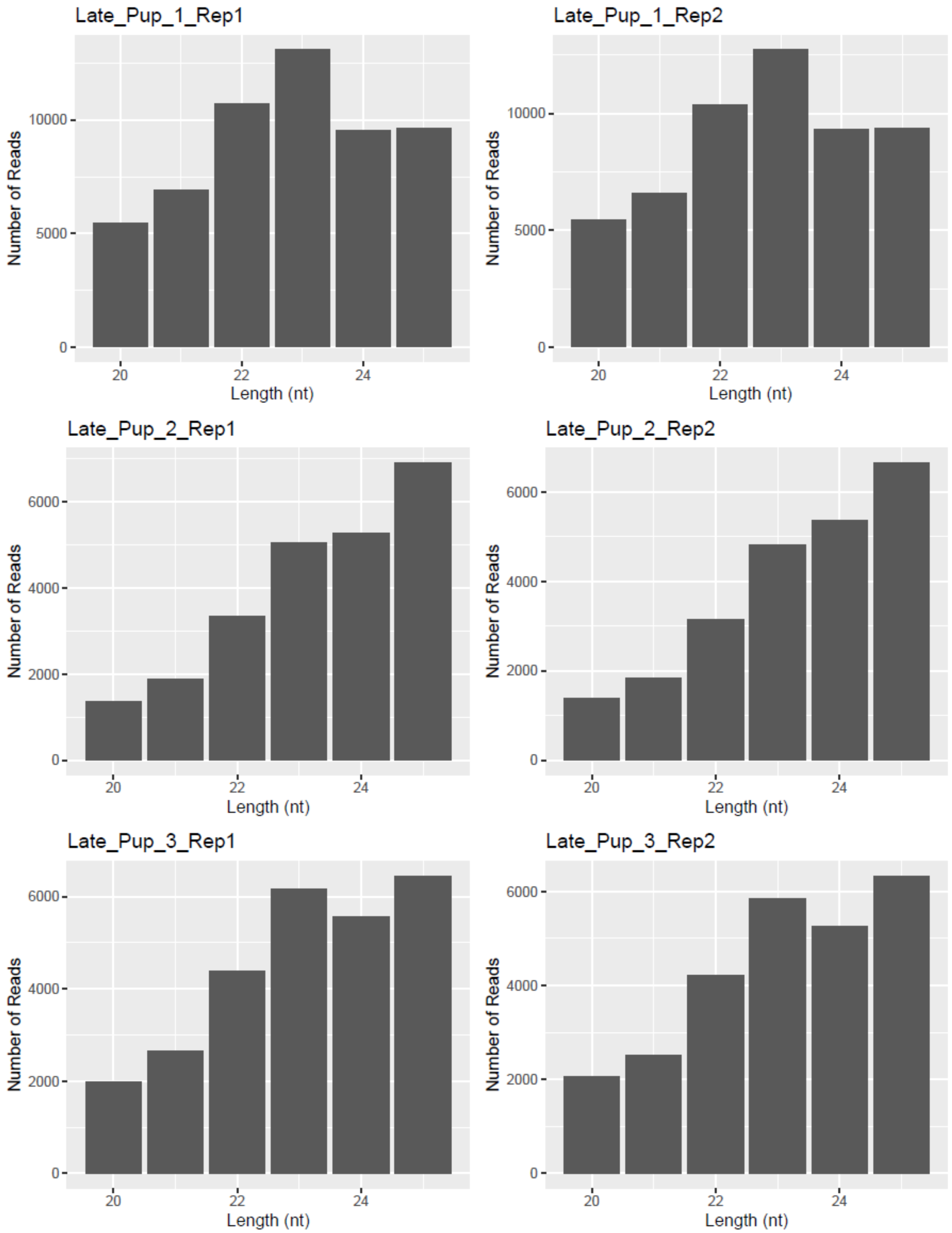

Figure S20: Distribution of number of reads by read length (20-25 nt) for Late intrapuparial samples

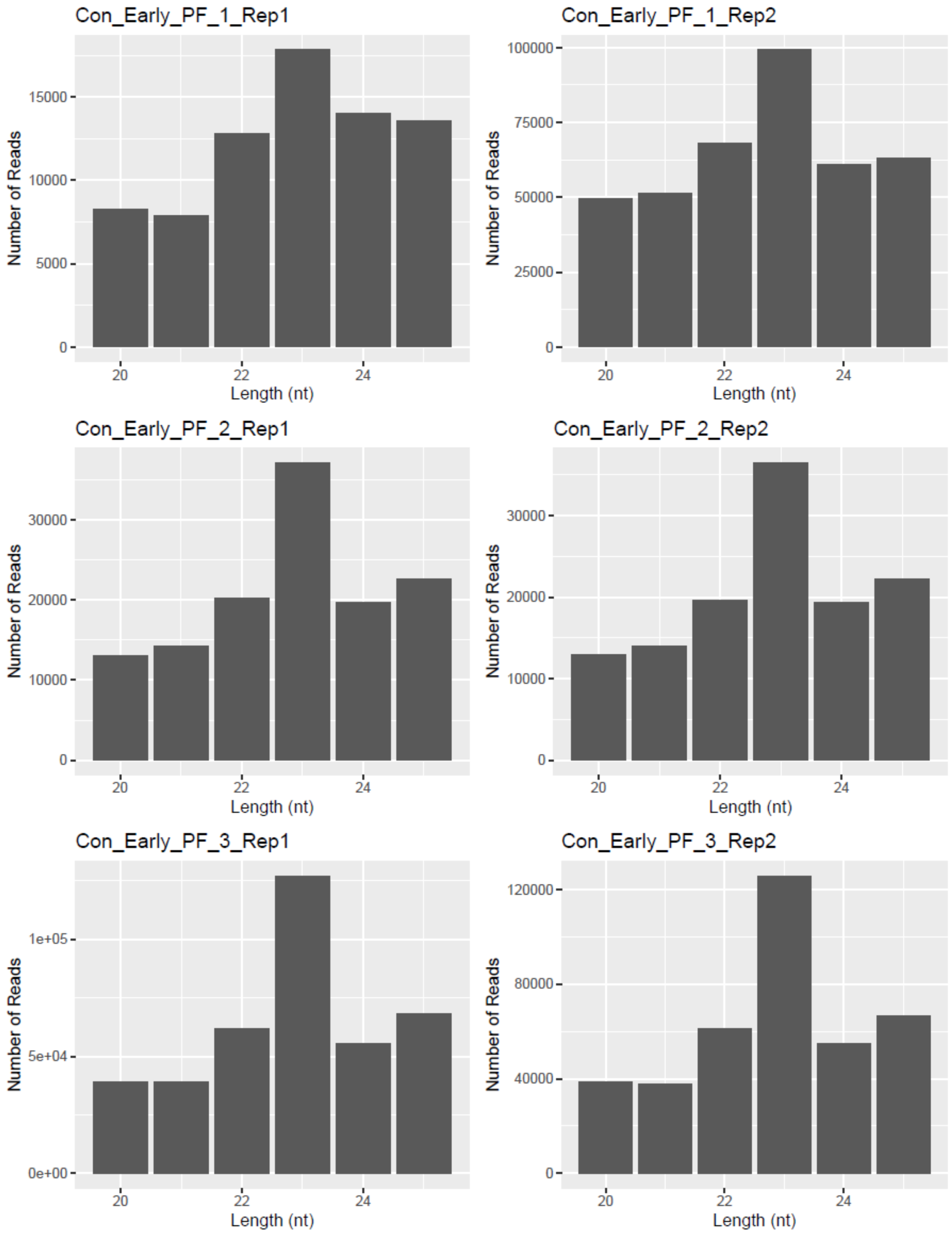

Figure S21: Distribution of number of reads by read length (20-25 nt) for Control early postfeeding samples

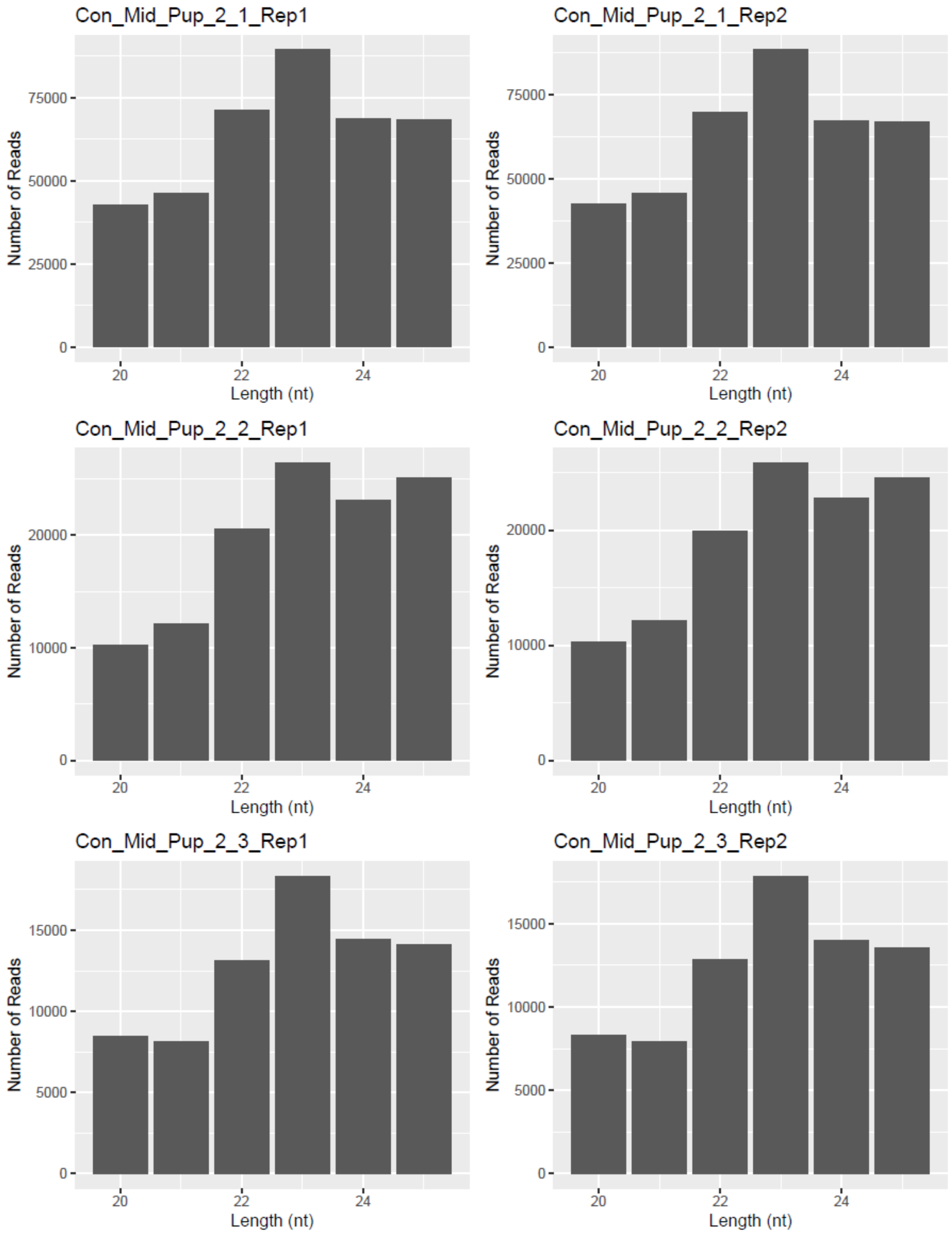

Figure S22: Distribution of number of reads by read length (20-25 nt) for Control mid intrapuparial 2 samples

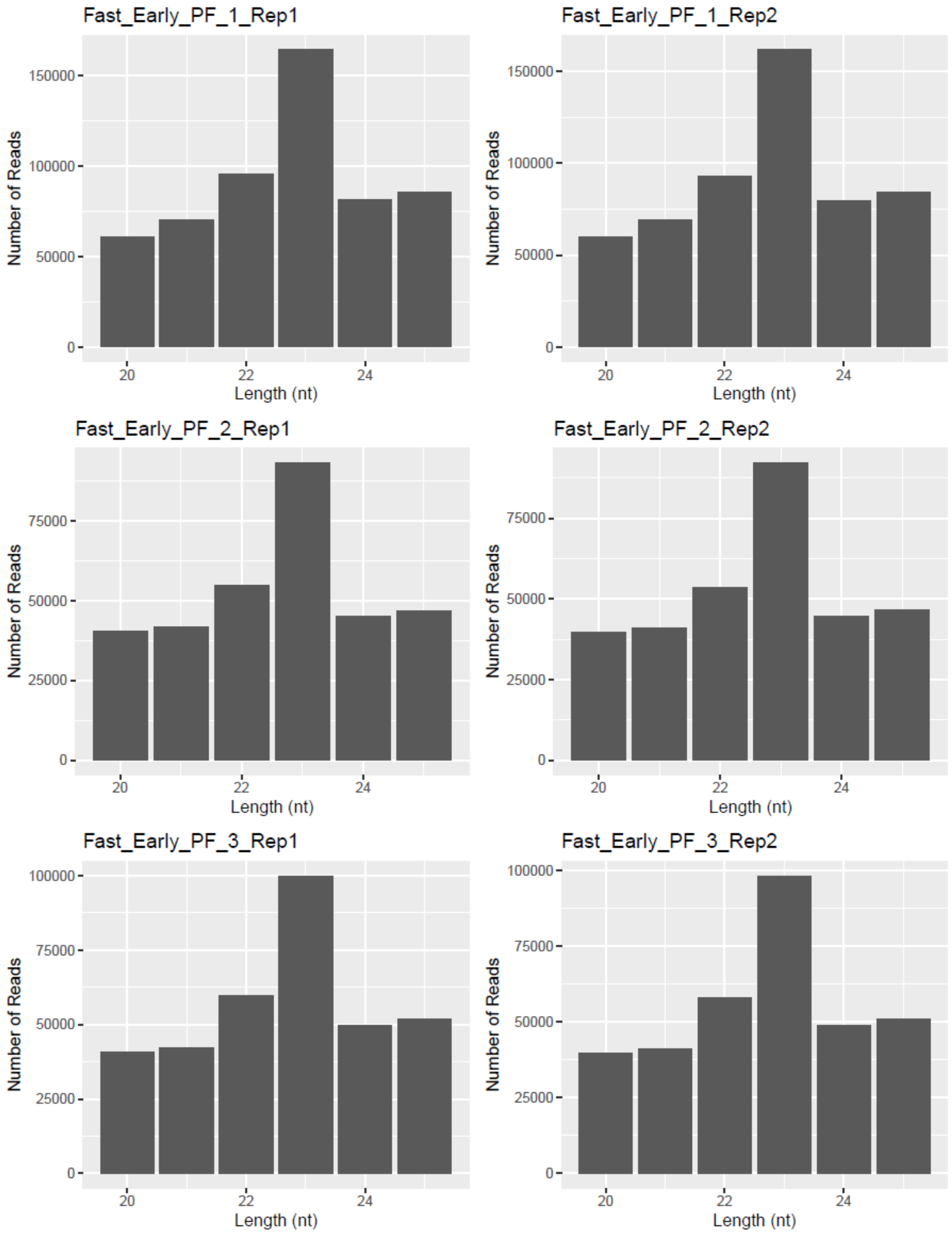

Figure S23: Distribution of number of reads by read length (20-25 nt) for Fast early postfeeding samples

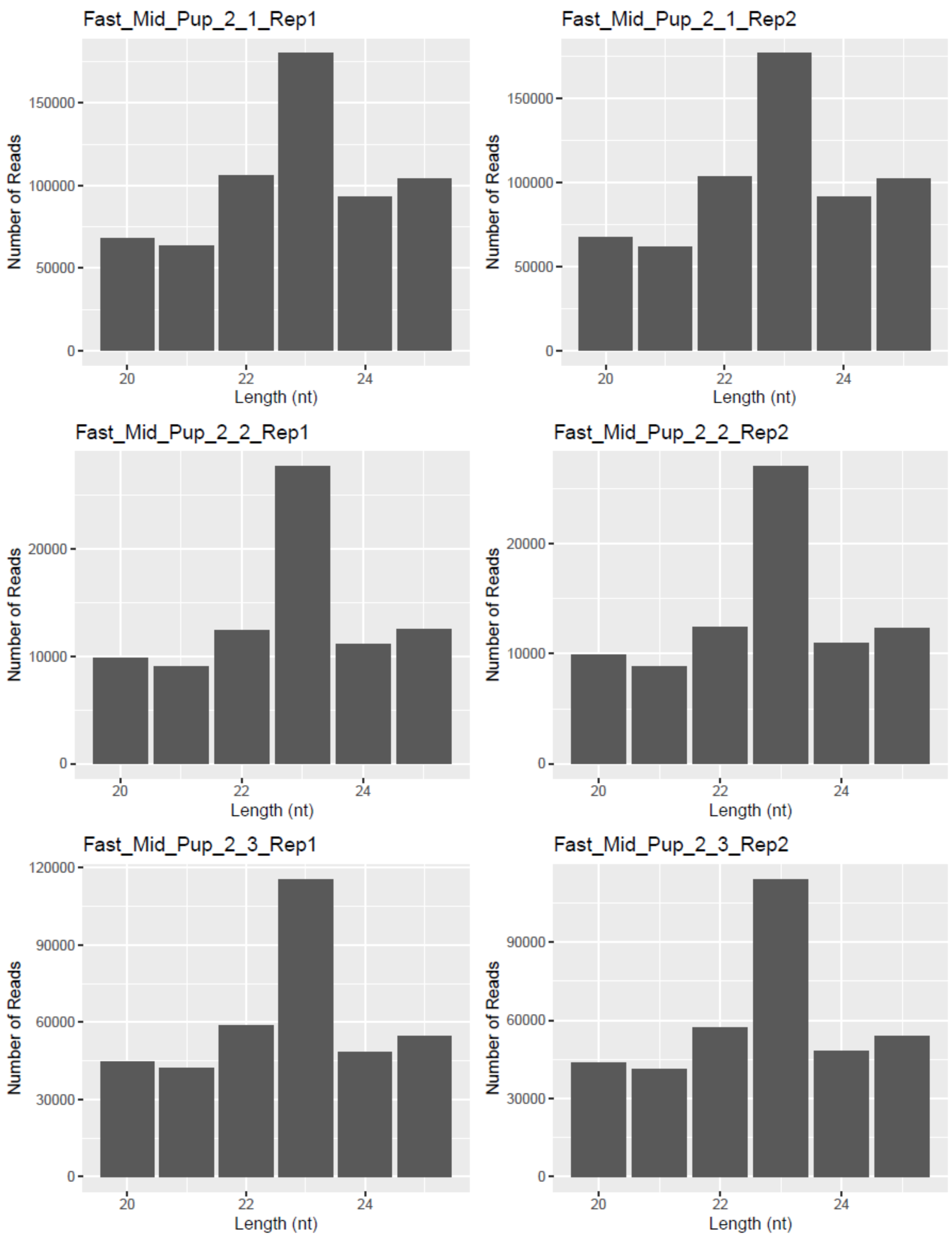

Figure S24: Distribution of number of reads by read length (20-25 nt) for Fast mid intrapuparial 2 samples

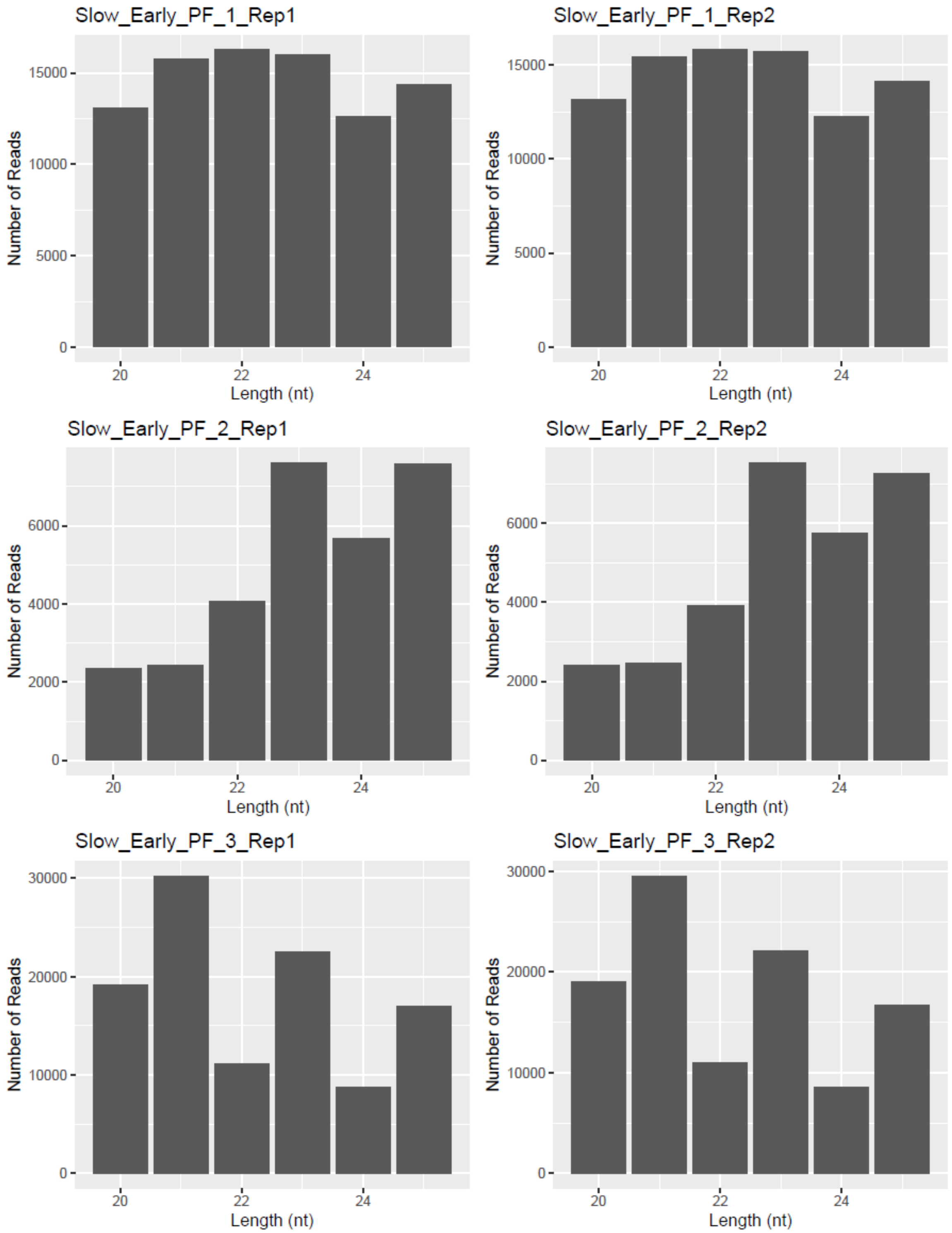

Figure S25: Distribution of number of reads by read length (20-25 nt) for Slow early postfeeding samples

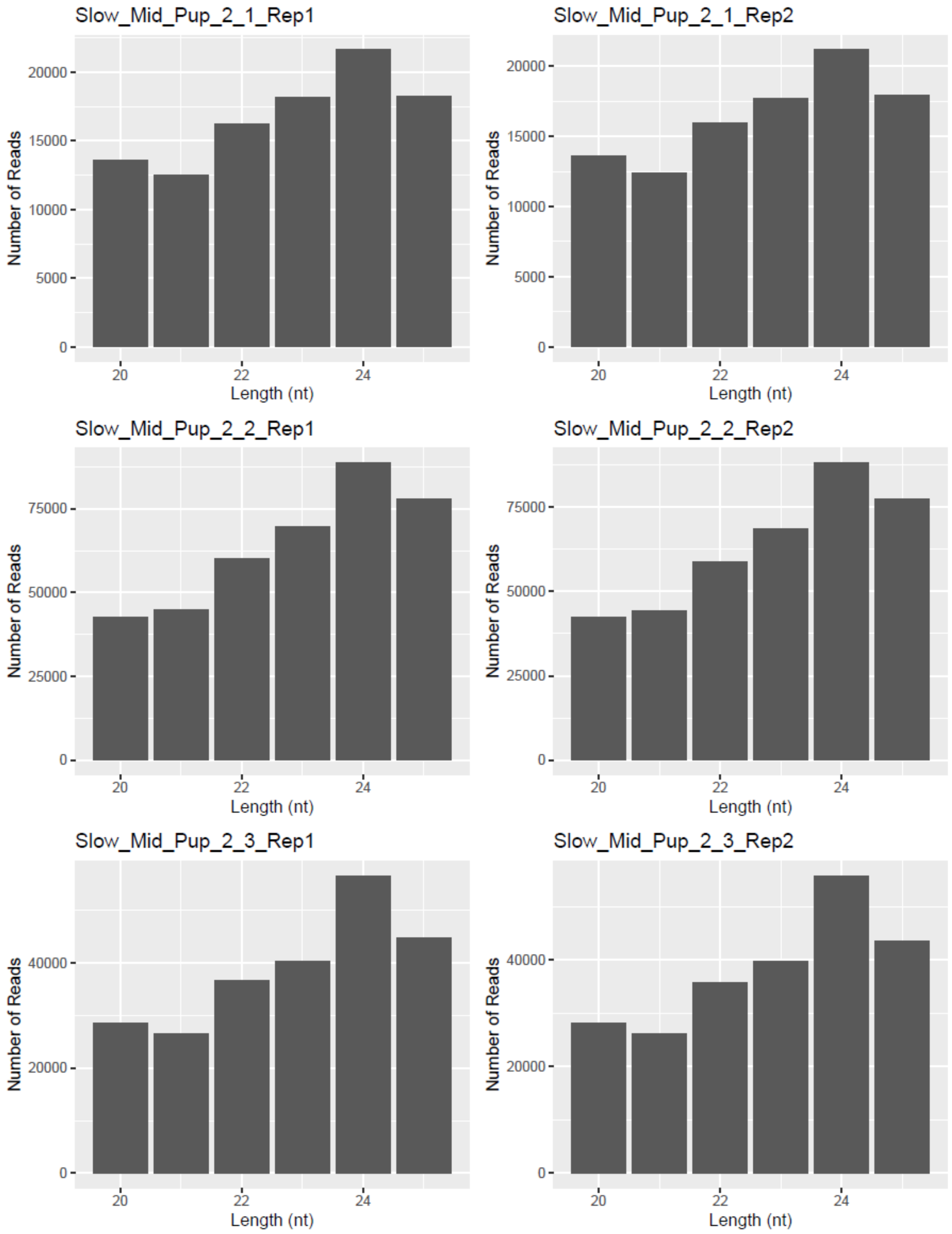

Figure S26: Distribution of number of reads by read length (20-25 nt) for Slow mid intrapuparial 2 samples

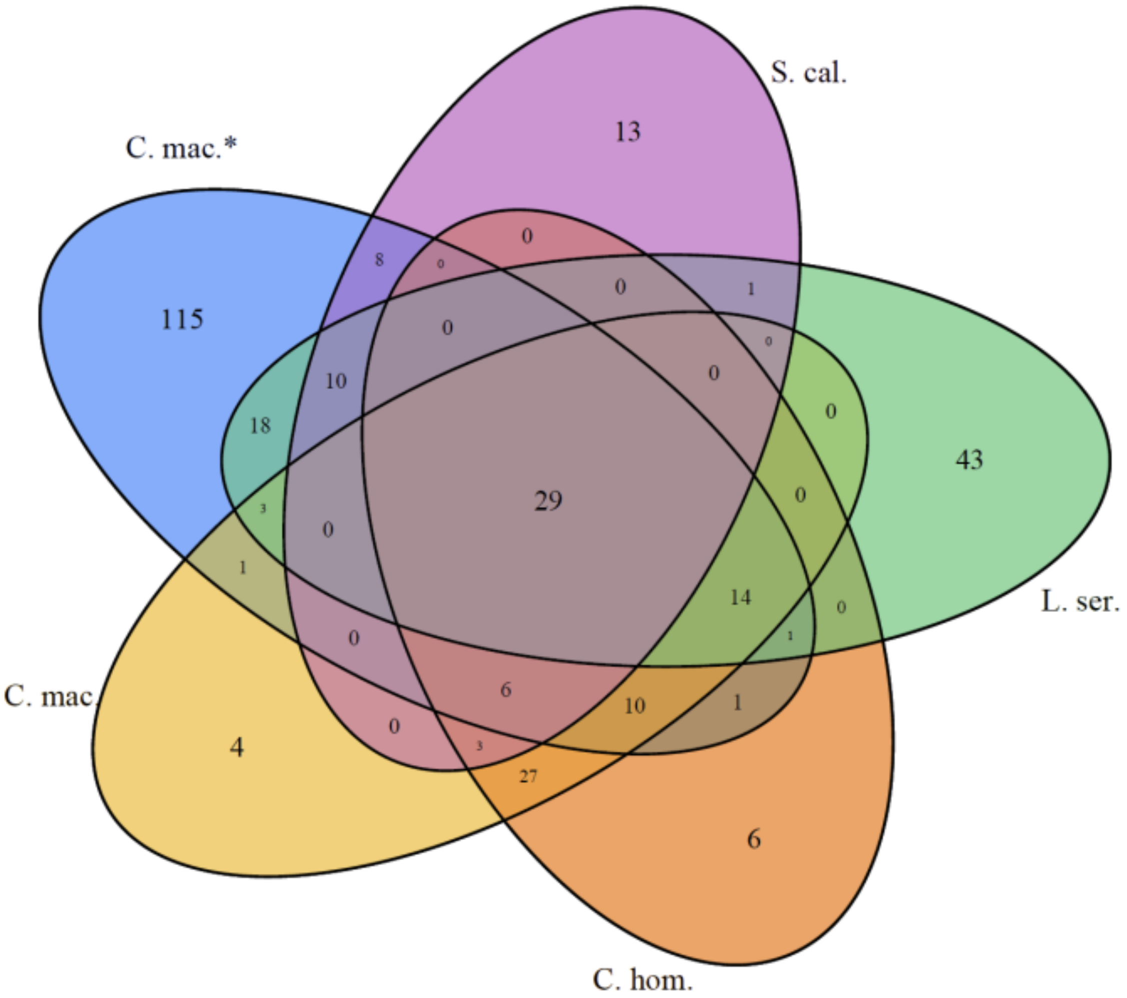

Figure S27: Venn diagram comparing number of miRNA identified between calliphorid and muscid flies.

Numbers of shared identified miRNA from previous studies in C. macellaria (Paulo et al., 2017), C. hominivorax (Paulo et al., 2017), Lucilia sericata (Blenkiron et al., 2015), and Stomoxys calcitrans (Tuckow et al., 2013) were compared to C. macellaria from this current study (C. mac*) and visualized in a Venn diagram format. Of the 115 unique miRNA identified in this study 58 were mapped to vertebrate species.

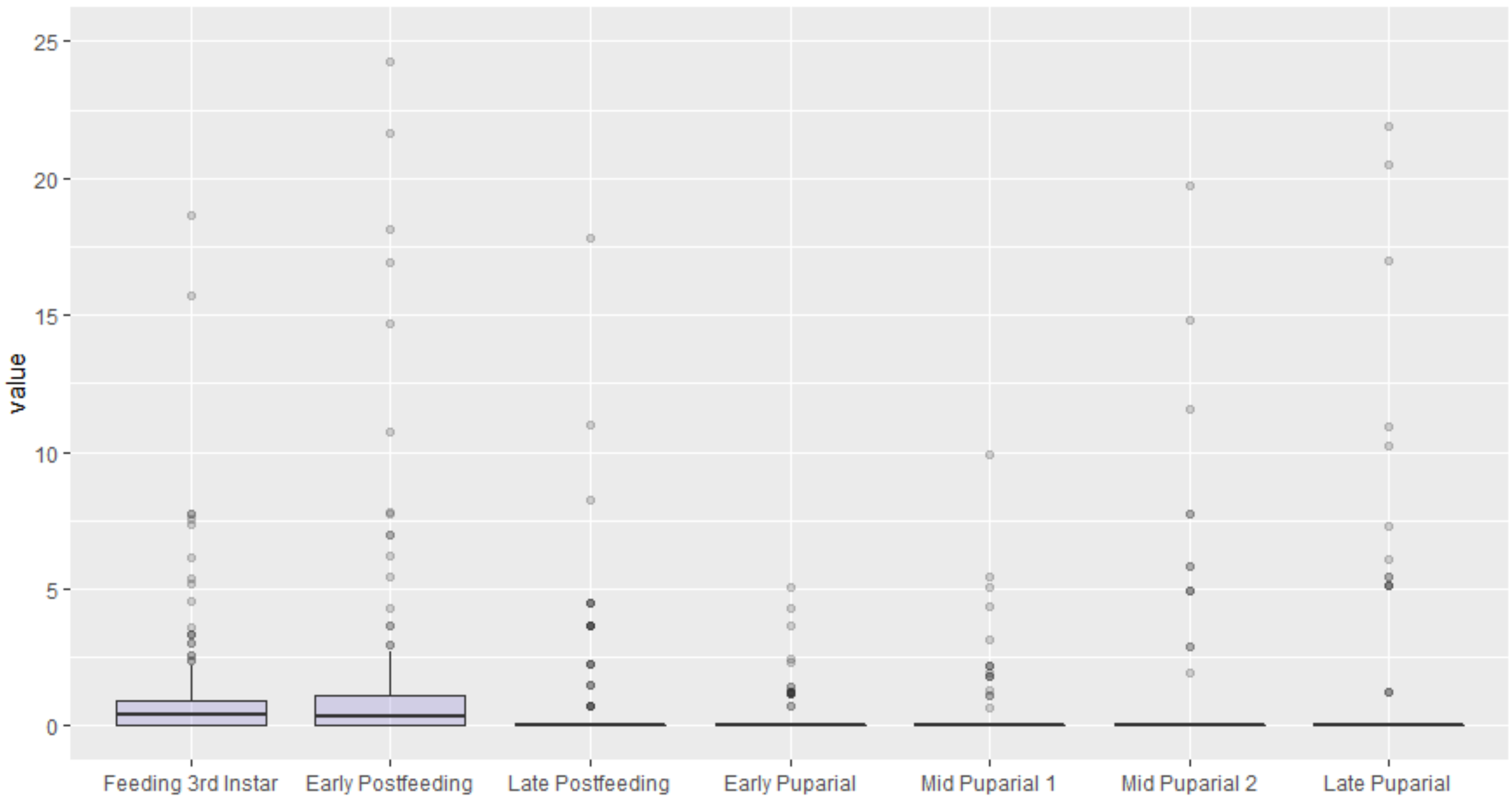

Figure S28: Vertebrate miRNA Expression levels show decrease in expression after early postfeeding larval stages. This decrease in sequence presence suggests that these miRNA are not being expressed in C. macellaria, rather are residual miRNA from the bovine liver meal. Information on vertebrate miRNA may be important for determining what the meal was, but may also assist in differentiating feeding and postfeeding larval stages.

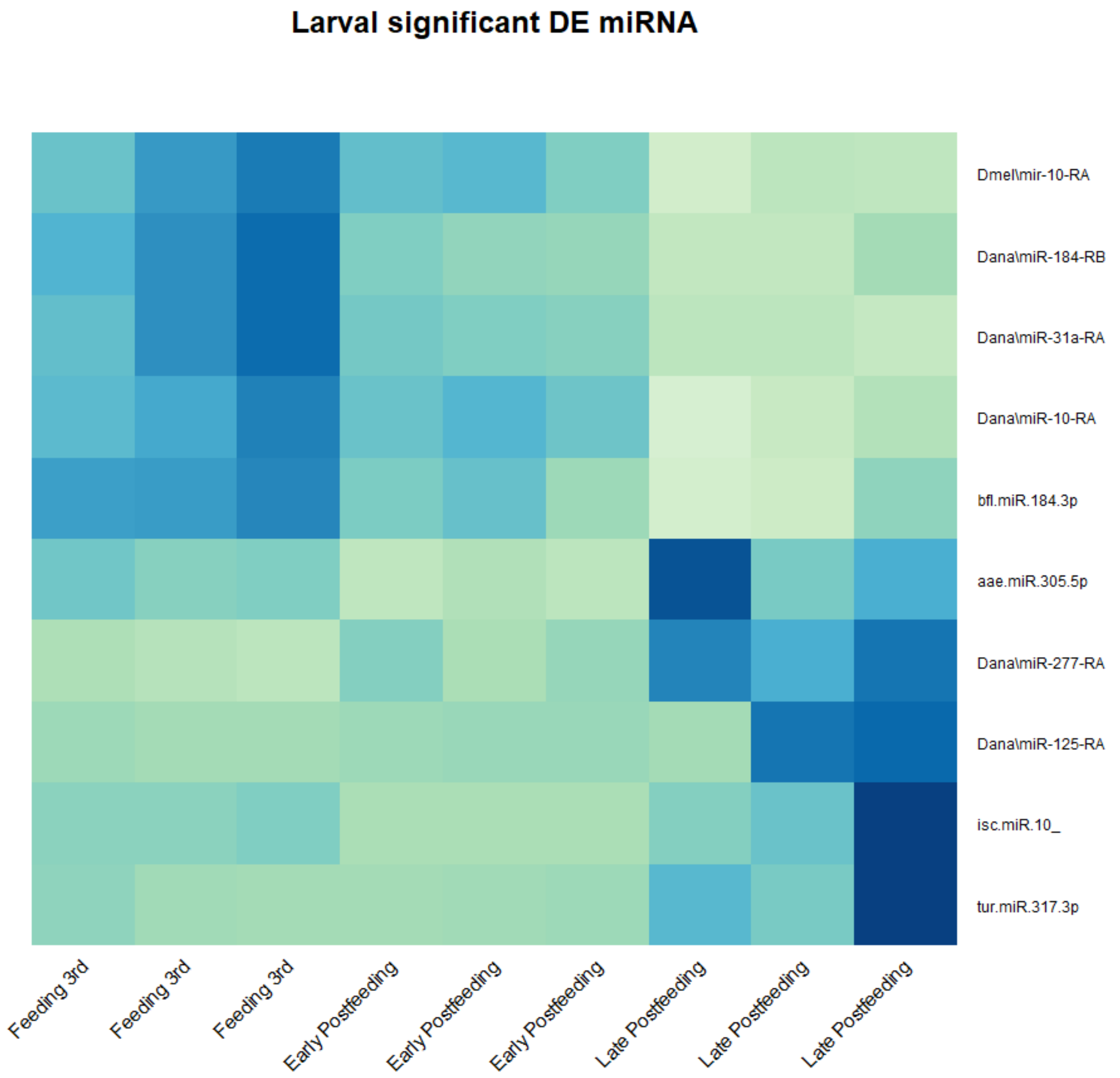

Figure S29: Heatmap of significantly differentially expressed miRNA across larval development in wild-type samples. Row names correspond to name of miRNA in Flybase or miRbase databases. Darker coloration indicates higher expression

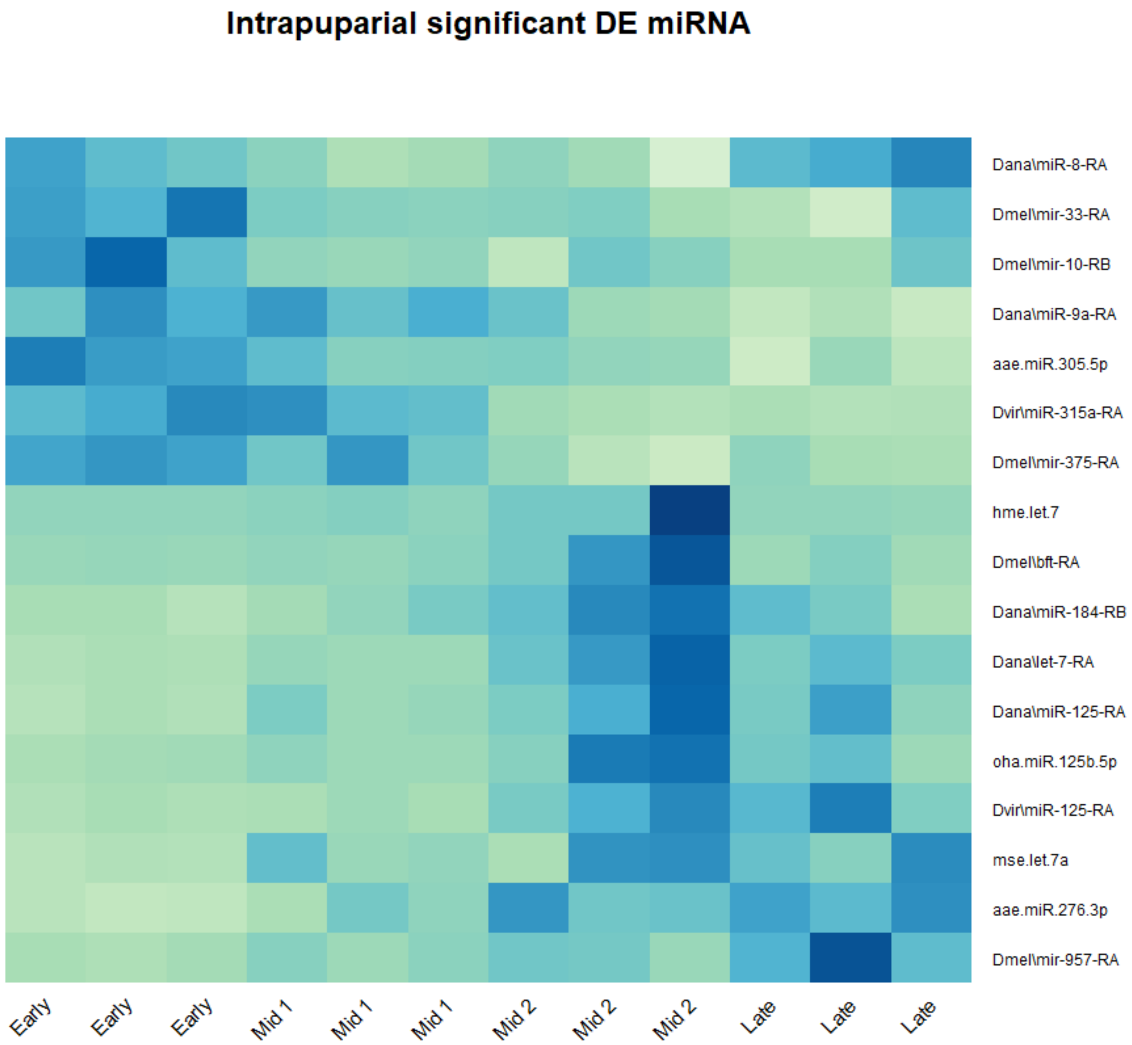

Figure S30: Heatmap of significantly differentially expressed miRNA across intrapuparial development in wild-type samples. Row names correspond to name of miRNA in Flybase or miRbase databases. Darker coloration indicates higher expression

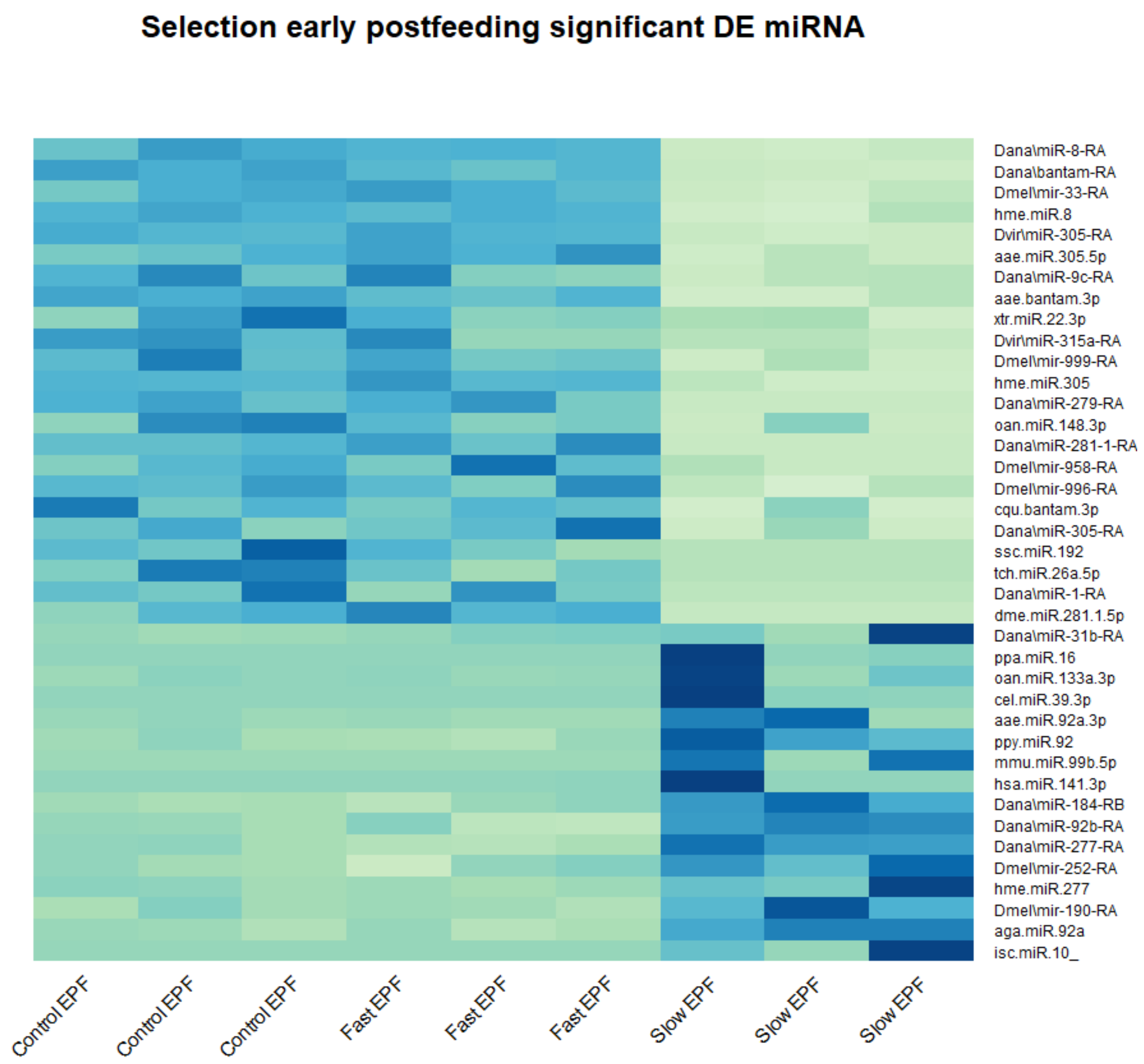

Figure S31: Heatmap of significantly differentially expressed miRNA across development time selected early postfeeding 3rd instar larval samples. Row names correspond to name of miRNA in Flybase or miRbase databases. Darker coloration indicates higher expression

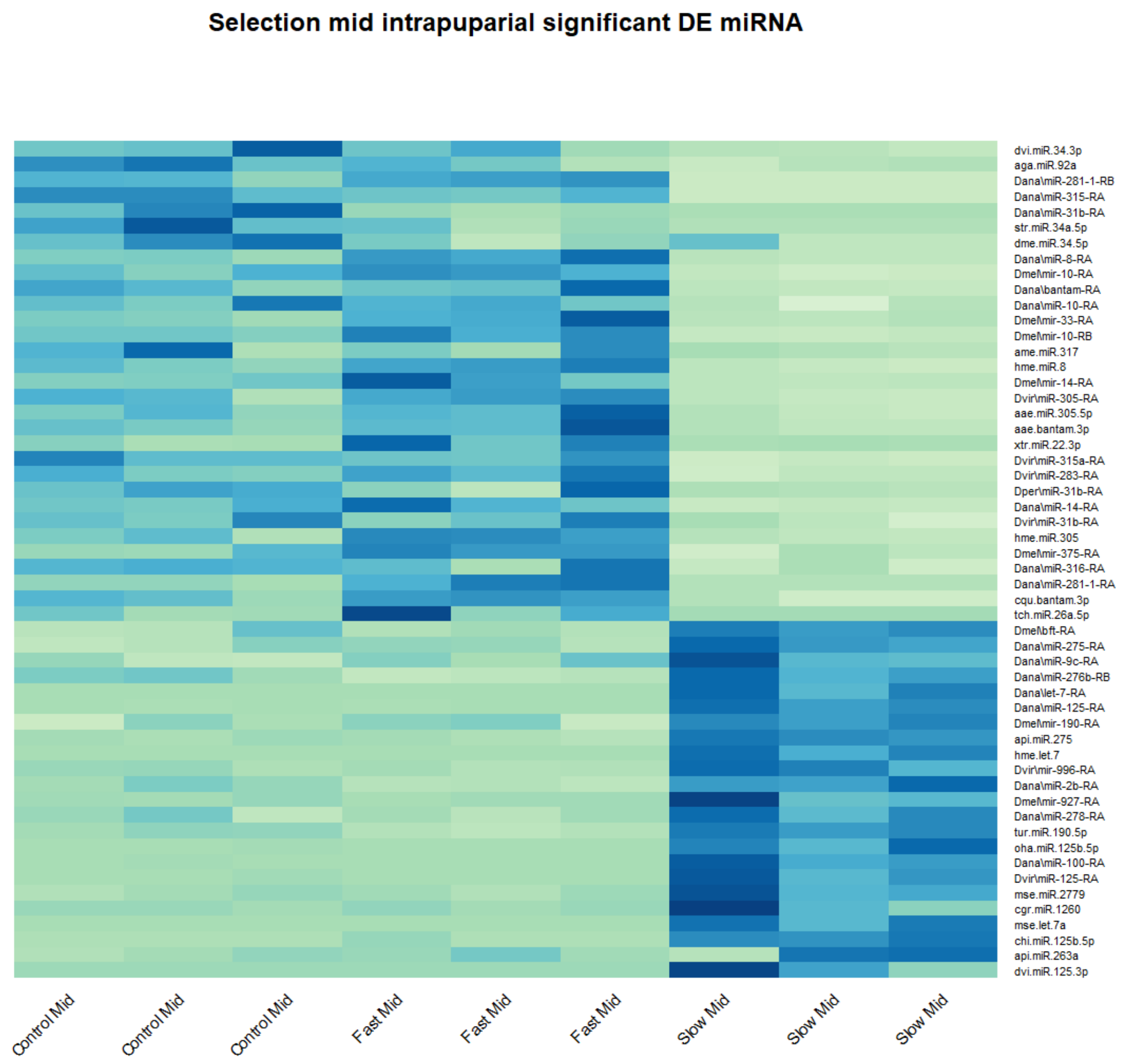

Figure S32: Heatmap of significantly differentially expressed miRNA across development time selected mid intrapuparial samples. Row names correspond to name of miRNA in Flybase or miRbase databases. Darker coloration indicates higher expression

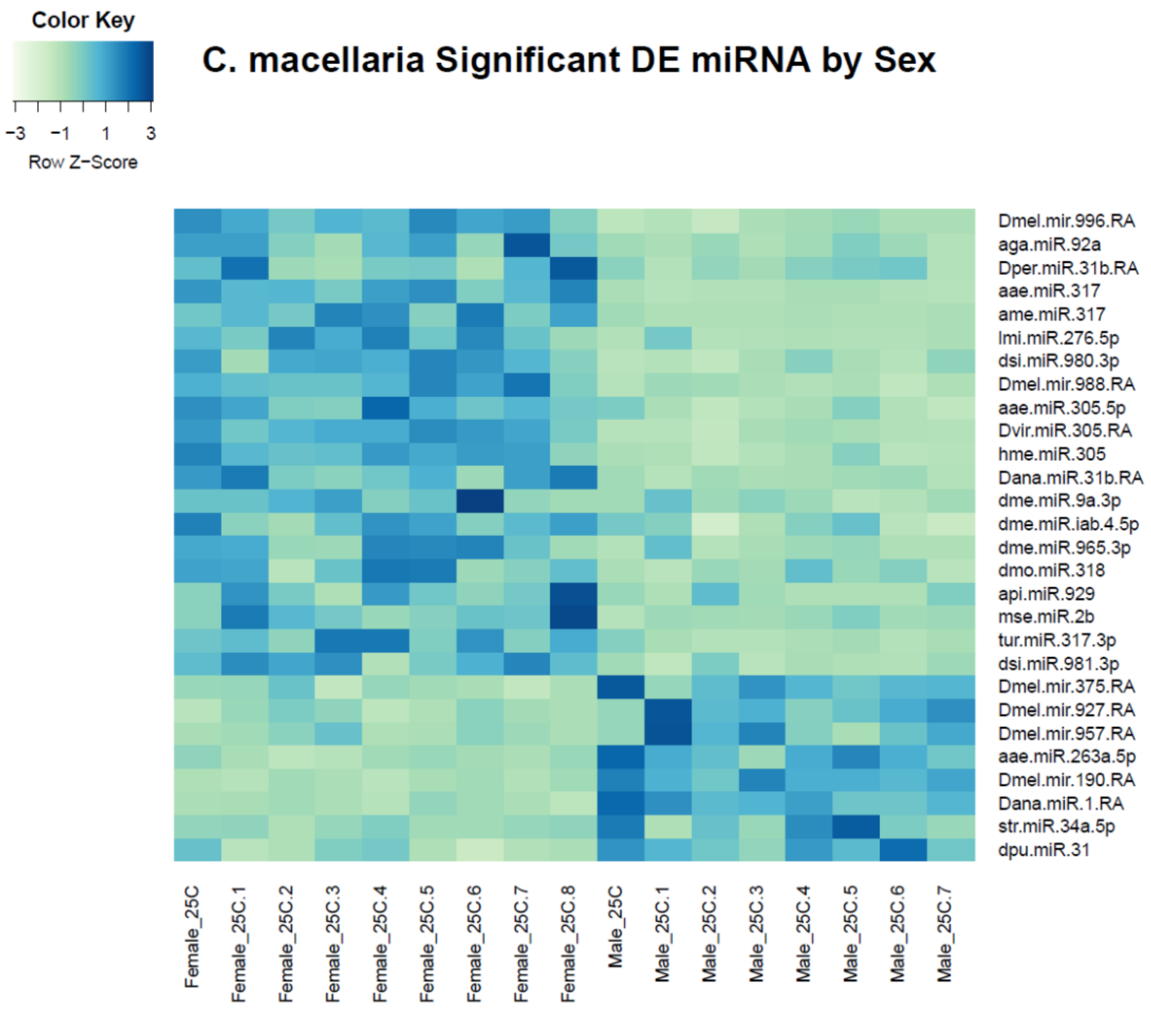

Figure S33: Heatmap of significantly differentially expressed miRNA at early intrapuparial stage between male and female samples.

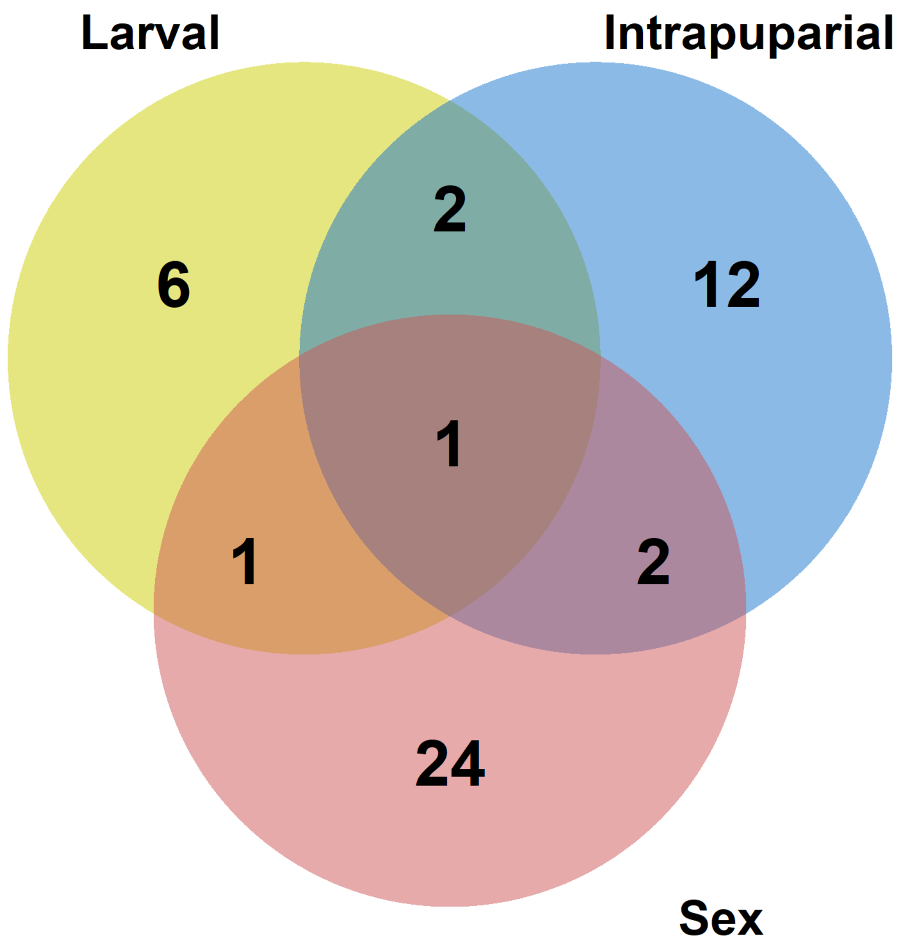

Figure S34: Venn Diagram of shared differentially expressed miRNA determined by DESeq2. Larval and Intrapuparial refer to differentially expressed miRNA in wild-type development samples

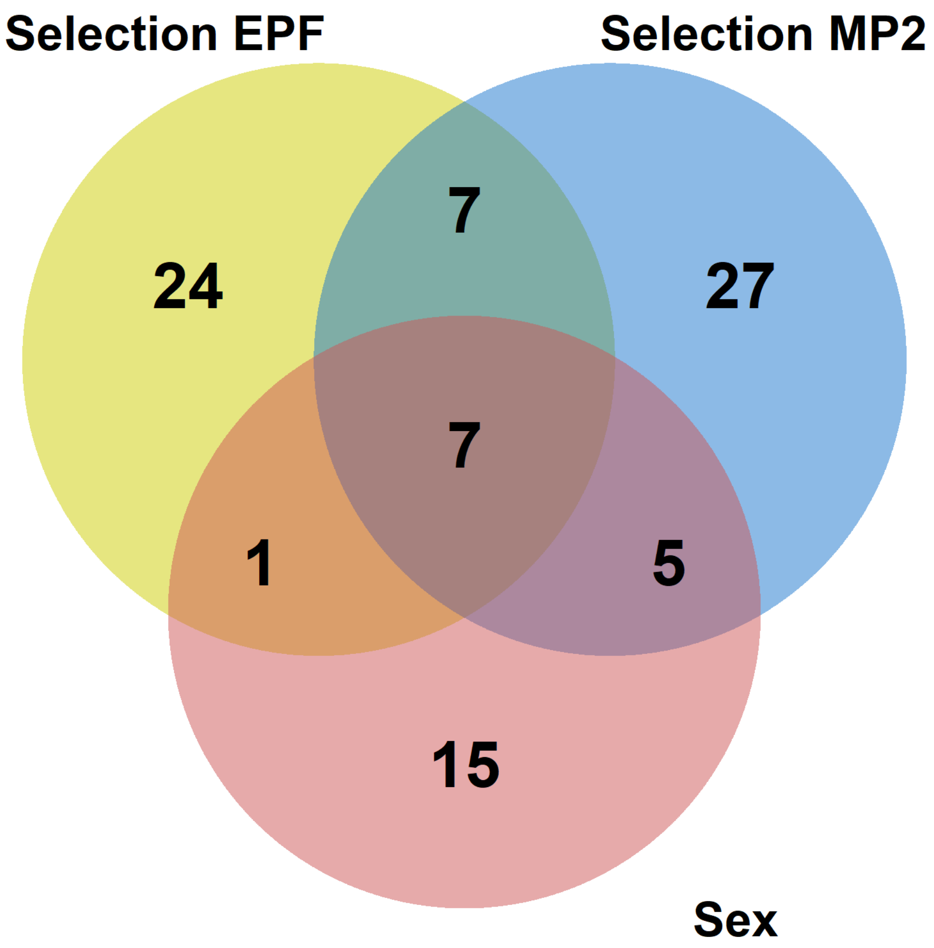

Figure S35: Venn diagram of shared differentially expressed miRNA determined by DESeq2 Development-time-selected samples EPF (Early postfeeding) and MP2 (Mid-intraPuparial 2) and Sex

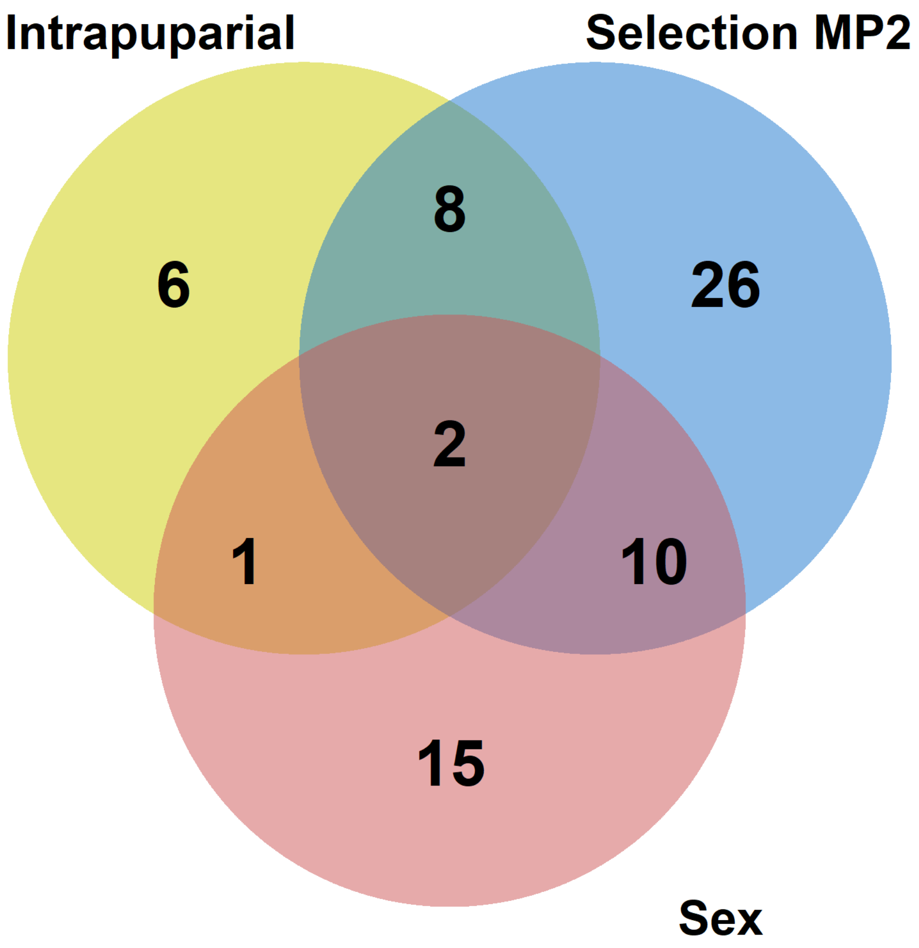

Figure S36: Venn Diagram of shared differentially expressed miRNA in intrapuparial samples: Wild-type intrapuparial, Development-time-selected mid-intrapuparial 2 samples, and sex

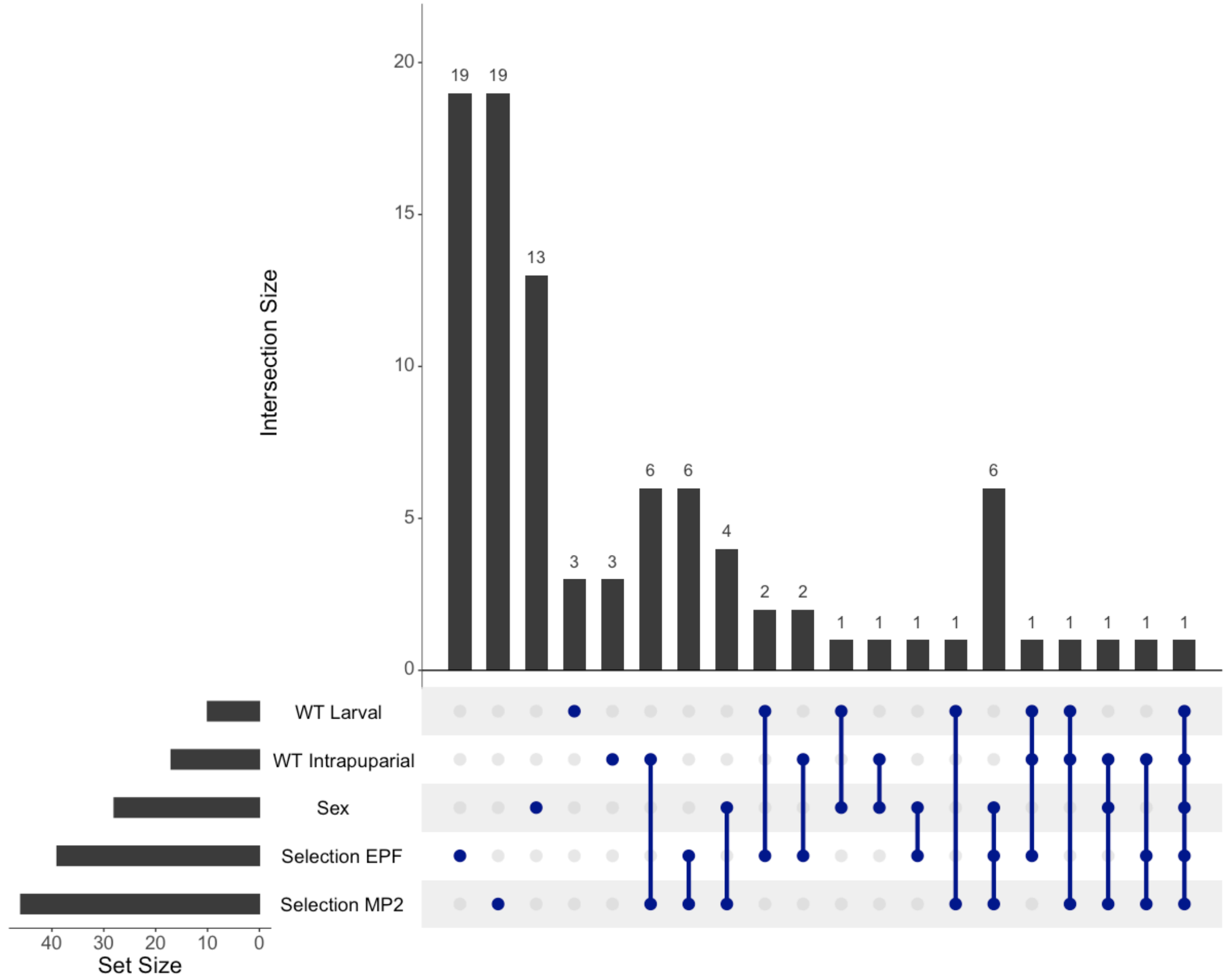

Figure S37: Upset plot of all shared differentially expressed miRNA (according to DESeq2) in larval, intrapuparial, selection (EPF and MP2), and sex specific samples. Dark dots correspond to groups being compared. Lines between dots correspond to the categories being compared. Bars on the intersection plot (top) correspond to the number of shared differentially expressed miRNA
